# Challenges and future trends in wearable closed-loop neuromodulation to efficiently treat methamphetamine addiction

**DOI:** 10.3389/fpsyt.2023.1085036

**Published:** 2023-02-23

**Authors:** Yun-Hsuan Chen, Jie Yang, Hemmings Wu, Kevin T. Beier, Mohamad Sawan

**Affiliations:** ^1^CenBRAIN Neurotech Center of Excellence, School of Engineering, Westlake University, Hangzhou, China; ^2^Institute of Advanced Technology, Westlake Institute for Advanced Study, Hangzhou, China; ^3^Department of Neurosurgery, Second Affiliated Hospital, School of Medicine, Zhejiang University, Hangzhou, China; ^4^Department of Physiology and Biophysics, University of California, Irvine, Irvine, CA, United States; ^5^Department of Neurobiology and Behavior, University of California, Irvine, Irvine, CA, United States; ^6^Department of Biomedical Engineering, University of California, Irvine, Irvine, CA, United States; ^7^Department of Pharmaceutical Sciences, University of California, Irvine, Irvine, CA, United States; ^8^Center for the Neurobiology of Learning and Memory, University of California, Irvine, Irvine, CA, United States

**Keywords:** closed-loop neuromodulation, wearable devices, methamphetamine addiction, EEG-fNIRS, multimodal, neuroimaging biomarkers, TMS technique

## Abstract

Achieving abstinence from drugs is a long journey and can be particularly challenging in the case of methamphetamine, which has a higher relapse rate than other drugs. Therefore, real-time monitoring of patients’ physiological conditions before and when cravings arise to reduce the chance of relapse might help to improve clinical outcomes. Conventional treatments, such as behavior therapy and peer support, often cannot provide timely intervention, reducing the efficiency of these therapies. To more effectively treat methamphetamine addiction in real-time, we propose an intelligent closed-loop transcranial magnetic stimulation (TMS) neuromodulation system based on multimodal electroencephalogram–functional near-infrared spectroscopy (EEG-fNIRS) measurements. This review summarizes the essential modules required for a wearable system to treat addiction efficiently. First, the advantages of neuroimaging over conventional techniques such as analysis of sweat, saliva, or urine for addiction detection are discussed. The knowledge to implement wearable, compact, and user-friendly closed-loop systems with EEG and fNIRS are reviewed. The features of EEG and fNIRS signals in patients with methamphetamine use disorder are summarized. EEG biomarkers are categorized into frequency and time domain and topography-related parameters, whereas for fNIRS, hemoglobin concentration variation and functional connectivity of cortices are described. Following this, the applications of two commonly used neuromodulation technologies, transcranial direct current stimulation and TMS, in patients with methamphetamine use disorder are introduced. The challenges of implementing intelligent closed-loop TMS modulation based on multimodal EEG-fNIRS are summarized, followed by a discussion of potential research directions and the promising future of this approach, including potential applications to other substance use disorders.

## 1. Introduction

Addiction is defined as a strong need to use a particular substance or engage in a specific behavior, often in spite of harmful consequences. Addiction not only causes personal health problems but can have severe social impacts (1, 2). The most common addictions involve alcohol, drugs, gambling, and smoking; and during the COVID-19 pandemic, the incidence of internet addiction increased owing in part to the limited availability of alternative activities allowed during quarantine (3). However, drug addiction or substance use disorder is perhaps the most severe example, and laws have been established internationally to ban the use, sale, transport, and promotion of specific drugs, including heroin, cocaine, methamphetamine (METH), amphetamine, and cannabis. When an individual first experiences the rewarding effects of drugs, the habit of drug-seeking develops (4). Thus, more of the substance is needed to maintain satisfaction, and the individual experiences an impulse to use the drug even though doing so is harmful to health (5). Followed by increased drug cravings and further resulting in executive dysfunction. These disrupted reward-related processes in the brain cause physical and mental problems. Physically, the immune, digestion, respiration, cardiovascular, and, in particular, neurological systems are often damaged by addiction to drugs (6, 7). Mental health problems include depression, anxiety, psychosis, violence, suicide, etc. (8). Addictions might be a multidimensional disorder that includes several subtypes with different neurobiological underpinnings. This might lead to emotional and behavioral dysregulation (9–11). The effects of dysregulation often associated with increased risk of suicide (9, 12). Moreover, disordered behavior resulting from addiction creates a severe economic burden on families and society (13). Long-term use and dependence typically results in a variety of maladaptive behaviors and negative outcomes, and the current efficacy of existing interventions is limited.

Methamphetamine (METH) has been among the most frequently misused drugs for the past two decades in Southeast and East Asia (14). This is largely because of the geographical proximity to production and trafficking resources (15). To treat and prevent misuse of METH, clinicians and researchers have studied the mechanisms of addiction (16, 17). There are various approaches to treatment, including detox, behavior therapy, and peer support (18), and behavior therapy administered *via* a series of cognitive behavior tasks is currently considered to be the most effective approach to METH use disorder (17). In terms of medical therapy, the search for effective medicines to treat METH dependence and addiction is a hot research topic in the pharmaceutical field (19, 20). However, no promising results have been found. During the treatment process and the follow-up stages after abstinence has been achieved, the most serious challenge in treating METH addiction is relapse. Once relapse occurs, abstinence becomes more difficult.

Conventional approaches to detect METH usage include analysis of sweat, saliva, or urine (21–23). In addition, hair analysis may be used (24). During abstinence, questionnaires are used to evaluate the results of treatment or the risk of relapse (25, 26). However, detection of METH usage *via* these conventional methods is too slow to prevent relapse. Moreover, interpreting the outcomes of abstinence using questionnaires is subjective and can be inaccurate. One mechanism to reduce rates of relapse would be to reduce the preoccupation with and perseverance on METH before these maladaptive thoughts lead to actions. Therefore, real-time monitoring of patients’ physiological signals during abstinence before or when cravings arise is an important goal (27, 28). Use of METH and other drugs results in long-lasting brain changes, which can manifest in changes in brain signals when later exposed to cues, drugs, and/or stressors (17). Desires and cravings for METH also cause unique activity patterns in the brain, which can be observed using neuroimaging techniques (29–31). Analysis of brain signals can thus be used to determine whether an individual is or will be experiencing strong desire for METH. Once these biomarkers for METH cravings have been detected, corresponding actions can be implemented to reduce the potential for relapse.

Various neuroimaging techniques have been used to study the influence of METH use on cognitive functions (32). Functional magnetic resonance imaging (fMRI) is a commonly used tool to investigate the changes caused by METH and the recovery process of brain structures during rehabilitations (33). However, the temporal resolution of fMRI is relatively low, and its accessibility is limited. Other non-wearable neuroimaging techniques, such as positron emission tomography (PET) and magnetic resonance spectroscopy (MRS), are also not suitable for real-time monitoring. To achieve a timely response to the onset of craving when an individual is exposed to an environment where the desire for METH is triggered and to increase the efficacy of treatment, real-time monitoring techniques to record neural signal variations continuously are needed.

Electroencephalogram (EEG) and functional near-infrared spectroscopy (fNIRS) are promising tools for brain signal monitoring. Biomarkers in EEG signals have been explored in patients with METH addiction (33). Biomarkers specifically found in fNIRS signals recorded from METH-addicted participants have also been reported (34). Applying a multimodal EEG-fNIRS neuroimaging technique has many benefits, including enabling better understanding of neural coupling mechanisms compared with analysis of both signals recorded simultaneously. However, few studies have investigated the use of concurrent EEG and fNIRS signals (35). Monitoring such biomarkers not only can confirm the effect of the drug on an individual but also can be used to quantify METH cravings and thus inform treatment (36).

Various intervention approaches are used to help individuals with drug addictions. The conventional treatments are psychological counseling, family support, and legal restriction. These interventions are often planned according to a regular schedule with a set frequency. Family and social support often depend on the willingness and availability of others. Legal restrictions are often applied too late, when drug compulsive use is already established. Achieving abstinence from drug use is a long journey with a need for high self-motivation as well as external influences and legal constraints. Therefore, successful abstinence is a challenge. In contrast to the passive methods mentioned above, neurostimulation has the potential to alter brain activity to reduce cravings for drug use (37). Neuromodulation involves providing electrical, magnetic, optical, or ultrasound stimulation to the specific cerebral locations to interfere with neuron activity (38). Various modulation approaches have been shown to help with the management of neurological disorders such as addictions, resulting in alleviation or improvement in the clinical symptoms of the disorder (39).

**FIGURE 1:**
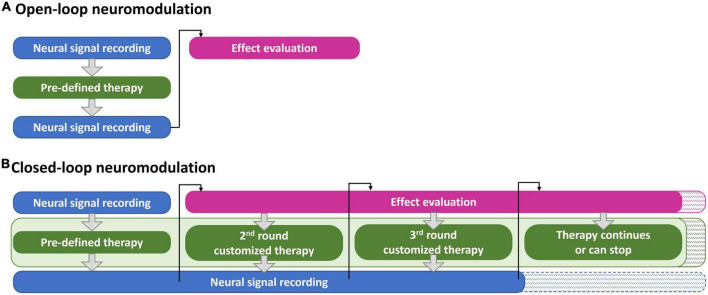
The workflow examples of **(A)** an open-loop neuromodulation system and **(B)** a closed-loop neuromodulation system for METH addiction treatment.

Neuromodulations exists as both invasive and non-invasive types. Deep brain stimulation (DBS) is the most frequently reported invasive neuromodulation solution for drug addiction (40). However, invasive devices can cause inflammation, limiting the feasibility of their long-term use. Therefore, non-invasive types are preferred owing to their wearability and accessibility. Commonly employed non-invasive neuromodulations for METH addiction include transcranial direct current stimulation (tDCS) and transcranial magnetic stimulation (TMS) (41, 42). tDCS changes the excitation states of neurons using low-dose direct currents (43). TMS modulates neuronal activity *via* a local current generated by magnetic fields of coils placed close to the scalp (44). Compared with tDCS, TMS has been shown in one study to achieve a longer and more stable effect against relapse in METH addiction, likely owing to the deeper stimulation depth and more precise targeting area (45). More important, TMS has been approved by the US Food and Drug Administration as an approved therapy for neurological diseases. However, at present, most commercially available TMS devices are bulky, reducing the accessibility of the treatment. Fortunately, applying TMS treatment remotely can increase the impact on the outcome. Therefore, miniaturization of TMS is an important goal. eNeura proposed a handheld TMS device to treat migraine (46). REMED introduced the first compact repetitive TMS (rTMS; using a train of repetitive magnetic pulses) device to initially treat major depressive disorder (47). However, compared with other wearable neurosignal-monitoring devices, such as EEG and fNIRS, portable TMS devices are not user-friendly because of their size and weight. Moreover, the current protocols for TMS therapy for METH addiction are based on the results of previous studies, and the protocol assigned to a patient may not be appropriate for that specific person. In addition, the therapy is often carried out on a regular schedule owing to the limited availability of the devices and device operators. To increase the success rate of TMS therapy for drug addiction, a wearable and compact closed-loop system to accurately provide a timely and appropriate treatment protocol to each person according to their needs is required (Figure 1) (48). Conventional open-loop neuromodulation validates the effects by comparing the related parameters before and after the interventions. No timely adjusted neuromodulation protocols can be applied based on the comparison results. In contrast, a closed-loop neuromodulation system can launch a new round of treatment with the optimal protocols for instant abnormal neurological disorders. The closed-loop system can help to determine the time when the treatment might can end.

To achieve effective treatment for METH addiction, three key elements are required for an intelligent closed-loop TMS neuromodulation system based on multimodal EEG-fNIRS measurements: (1) an appropriate measurement protocol for multimodal EEG-fNIRS monitoring, (2) intelligent signal-processing strategies, and (3) customized, user-friendly, wearable TMS devices. Section “2. Materials and methods” introduces the materials and methods of conducting the literatures collection for this review. Section “3. Detection and monitoring techniques for METH addiction” of this manuscript reviews the available techniques for physiological monitoring to detect drug use and addiction, with a particular focus is on wearable EEG and fNIRS neuroimaging approaches. In section “4. Biomarkers of neuroimaging techniques,” biomarkers in EEG and fNIRS recordings to identify METH addiction and the progression of recovery during abstinence are discussed. In section “5. Neuromodulation treatments for METH addiction,” the most common neuromodulation treatments for METH, tDCS, and TMS are introduced, and research supporting the efficacy of these treatments is summarized. The promising future of the application of intelligent closed-loop TMS modulation based on multimodal EEG-fNIRS for METH addiction is summarized in section “6. Challenges and future trends in treatment of METH addiction,” and the challenges to be overcome to achieve an optimized closed-loop system are discussed.

## 2. Materials and methods

We did not conduct a systematic review since this type of review is less common in engineering than in the medical and public health fields. This review aims to summarize the challenges and propose a future trend of a wearable closed-loop neuromodulation system for METH addiction treatment from an engineering point of view based on the available evidence. Our review provides insights into combing the three key elements, biomarkers, real-time signal analysis approach and neuromodulations, of the proposed closed-loop system. We do not aim to find a fixed answer to a specific question or an optimal medical therapy as a standard systematic review does. Neither needs all available evidence to support the concept of the wearable closed-loop system. In addition, the need to reduce the total bias and quantify (statistical analysis) the available results is not the top priority. For building a biomedical system, interdisciplinary knowledge is needed. Therefore, a systematic review might not be the best approach to convey our perspectives.

Since this review contains a wide subtopic, the key words used for searching the published journal papers are introduced in this section. The databases “Web of Science” and “Google” were used. For section “4.2. Biomarkers of EEG and fNIRS in METH addiction,” key words used to search the EEG biomarkers in METH addiction were EEG or Electroencephalography or Electroencephalographic and Methamphetamine or Meth. The keywords used to search fNIRS biomarkers in METH addiction are functional near-infrared spectroscopy, fNIRS, NIRS, and Methamphetamine or Meth. The validated papers suggest potential EEG or fNIRS biomarkers to distinguish the subjects with METH use disorder from healthy ones. In addition, articles that provide biomarker information to classify the subjects of METH use disorder receiving different treatments or at a different phase of abstinence are included in this review article. For section “5.1. tDCS for methamphetamine addiction,” key words applied for searching are transcranial direct current stimulation or tDCS and Methamphetamine or Meth. For section “5.2. TMS for methamphetamine addiction,” keywords used to explore the related studies are transcranial magnetic stimulation or TMS or theta burst stimulation and Methamphetamine or Meth. Only the studies with solid conclusions that certain tDCS or TMS protocols are helpful to treat patients with METH use disorder are included in this review article. No matter the validation approaches for the outcomes of neuromodulation. The techniques include self-rating scales, questionnaires, cognitive tasks, physiological signals, or neuroimaging. For all the found literature, only those that conducted the experiments on humans are reported in this review article.

## 3. Detection and monitoring techniques for METH addiction

Several approaches can be used to determine if an individual meets the criteria for diagnosis of a dependence or addiction. Subjectively, questionnaires are highly accessible and easily administered to evaluate the condition of drug addiction. Also, various methods are available to quantitatively detect physiological parameters of patients who use illicit drugs or suffer from drug addiction. Some methods detect drugs in biological fluids, while others detect neurological signals. These approaches are discussed in detail in the following sections.

### 3.1. Questionnaires

Various questionnaires have been developed for use before a person becomes addicted to drugs and during abstinence to predict the likelihood of relapse. The Inventory of Drug-Taking Situations is a questionnaire to judge the risk of drug addiction based on everyday situations. Abuse of drugs can be screened for using the assessment tools suggested by the National Institute on Drug Abuse (49). Among the suggested questionnaires, the most popular choice to examine an individual’s involvement with a variety of drugs is the Drug Use Questionnaire or Drug Abuse Screening Test (50, 51). To evaluate drug addiction over time, the Desire for Drug Questionnaire can be used to rate instances of cravings for drugs, whereas the Obsessive Compulsive Drug Use Scale evaluates cravings over a period of time (52). Regarding the effects of the drugs, the Visual Analog Scale (VAS), a visualization scale, is helpful for quantifying levels of craving for drugs. In addition, the Addiction Severity Index is used to evaluate overall issues, from personal to family and society, in the context of drug addiction (53).

As drug addiction affects has both physical and psychosocial effects, scales that characterize anxiety, depression, or impulsivity resulting from drug abuse are often used to obtain a broad view dependence and substance abuse. These scales include the 21-item Beck Anxiety Inventory, 21-item Beck Depression Inventory, and 30-item Barratt Impulsiveness Scale-11 (54). For the assessment of drug abstinence, the Drug Abstinence Self-Efficacy Scale is available (55). During the withdrawal period from drug use, the Subjective Opioid Withdrawal Scale, for example, can be used to check the symptoms of withdrawal (56), and the Methamphetamine Withdrawal Questionnaire was developed to specifically evaluate METH withdrawal-related symptoms (57). The Risk of Relapse Assessment Scale can be used to determine the possibility of relapse (26), while another option is the Stimulant Relapse Risk Scale (58). Furthermore, the Time to Relapse Questionnaire has been proposed to distinguish two types of relapses, with or without forewarning, to enable better treatment (59, 60).

Although questionnaires are the most convenient and broadly accessible approach for a variety of drug addiction-related applications, the results of the scales have limited reliability and accuracy. As the results of these scales are based on the answers of the respondents, the results exhibit individual variations and are influenced by the attitudes and conditions of the participants (61). Moreover, clinicians are often needed to draw conclusions about the severity of addiction based on the results of the scales and consultations. These limitations restrict the scope of application. Therefore, questionnaires often need to be combined with physiological tests to strengthen their conclusions about addiction, withdrawal, abstinence, or relapse.

### 3.2. Conventional detection techniques and emerging wearable techniques

In addition to questionnaires, saliva, urine, and blood tests are conventional ways to quantitatively detect drug use (22). Nail and hair also contain evidence of drug use with a detection window lasting from weeks to months (62, 63). Moreover, breath analyzers can be used to detect the drug in the breath (64). However, the time windows of the above conventional methods are limited. Moreover, they cannot provide real-time results owing to the time-consuming nature of the required examination and analysis procedures. Emerging wearables are promising options to detect biomarkers in real-time (27, 65), most commonly using sweat to screen for drugs using electrochemical techniques (66). Other body fluids containing drugs are saliva and tears. However, it is sometimes difficult to obtain sufficient body fluids for accurate sensing. In addition to electrochemical sensing of body fluids, wearables can detect other physiological parameters to predict or determine addiction to drugs: these include electrocardiographic (ECG) parameters, heart rate variability (HRV), breath rate, and skin conductance response/galvanic skin response (SCR/GSR). Both heart rate (HR), determined by the R-R interval of ECG signals recorded from a chest band and breath rate increase with increasing dosages of cocaine (67). Other parameters available in ECG waveforms for drug addiction evaluation are the QT, PR, QRS, and QTc intervals and the height of T waves, as summarized in previous work (67). Regarding the morphology of the breathing waveform, the time and depth of inhalation and exhalation, and respiration duration are features used to predict drug-seeking and craving for drugs. Furthermore, a wristband is another option to identify the use of cocaine based on recorded skin temperature, heartbeat, motion, and SCR (28, 68). HRV can be used to evaluate stimulation in individuals with METH addiction *via* virtual reality (VR) (69) and SCR increases in patients with METH addiction when receiving specific cues (70); importantly, longer use of METH results in a stronger physiological reaction to the cues. Owing to the ease of access and user friendliness of wristband devices for ECG recording, the effects of aerobic exercise on the HRV parameters of patients with METH addiction have been investigated (71). HRV can be further separated into high-frequency HRV and low-frequency HRV. In addition to the frequency domain, parameters can be derived from the recorded time domain of HR, including the standard deviation of normal-to-normal intervals, root mean square difference of the standard deviation, and percentage of beats that change by more than 50 ms compared with the previous beat.

In addition to ECG and HR, drug abuse has effects on pupil size (72). The features of ECG, GSP, and eye tracking that can indicate METH addiction are summarized in Tsai et al. (73). Wearables can be used not only to monitor physiological signals but also to track the psychological impact of drug addiction. For example, the information obtained from accelerometry and GPS location is useful in characterizing the cravings resulting from the addiction (74, 75). Owing to the large variety of the features available to diagnose drug addiction or the resulting psychological changes, machine learning (ML) has been introduced to increase the precision of the analyzed results; more information will be provided in section “6.1.2. Analysis of recorded neural signals.”

The wearable techniques presented in this section are used to measure the physiological reactions of the autonomic nervous system caused by drug usage. However, drug addiction also affects the central nervous system (7). We believe that the development of a closed-loop system for overall management of drug addiction would provide substantial value, regardless of whether patients are still using drugs, in the abstinence stage, or aim to reduce the rate of relapse rate. Application of this closed-loop system would rely on neuroimaging techniques, being the most straightforward approaches for brain studies, to identify and characterize the brain signatures of drug addictions.

### 3.3. Neuroimaging techniques

Whereas the wearable technologies discussed in the previous section can be used to monitor physiological changes in the body as relates to drug use and withdrawal, clinicians and researchers are also keen to learn how drug use influences the control center of the body, the brain. The process of developing an addiction includes several phases, including drug intoxication, craving, binging, and withdrawal with loss of self-control (76–78). A series of complex changes occur, including modification of brain structures as well as mental and physiological changes, resulting in various symptoms such as depression, impulsiveness, anxiety, aggression, and many other psychological problems (11). Consequently, a tool to monitor the response/effect of a neuromodulation treatment is a need for a closed-loop system. Neuroimaging techniques provide opportunities to monitor the overall process of the functional modifications of the central nerve system in various conditions (79). Therefore, they are suitable to study the mechanisms of neurological disorders (76, 80).

Neuroimaging approaches can be separated into two categories: those that can be used to examine the structural changes at different stages of drug dependence and addiction to understand the physiological mechanisms of addiction (30), and those that monitor the functional brain changes that occur as a consequence of addiction (77). MRI, MRS, and single photon emission computed tomography are used to inspect structures in patients with METH addiction (31, 32, 81). PET is used to study the impact of drug addiction on the brain at a molecular level, in order to find the optimal brain location for treatments (82). fMRI is a powerful tool to evaluate hemodynamic conditions after the neurons have been activated by specific tasks (83, 84). The studies investigating the relationship between METH use and cognitive function using neuroimaging techniques have been summarized elsewhere (32).

Although these neuroimaging techniques provide high spatial resolution, they have limitations. For instance, the devices required are costly and bulky, with low accessibility, and trained operators are needed to conduct the examinations. Moreover, MRI may not be suitable for patients with metallic implants, and the use of radioactive agents in PET limits the frequency of examinations. Furthermore, those with claustrophobia may find it difficult to participate the examinations owing to the spatially confined test environment that is needed. Low temporal resolution also restricts the application of real-time monitoring.

Wearable devices, being compact and easy to access, are appropriate for real-time monitoring of neural activity. Owing to their user-friendly implementation, wearable neuroimaging devices have been widely used to study the outcomes of various treatments, including the effects of exercise on parameters of drug addiction (85). Another example is the evaluation of cravings to predict the risk of relapse during abstinence. Real-time signals recorded in a natural environment provide more reliable information than could be obtained under stressful conditions using bulky equipment.

Electroencephalogram can be used to record brain cortical electrical activity *via* electrodes attached to the individual’s head. It is popular in research and clinical studies owing to the high temporal resolution of afforded by this approach. This benefit enables EEG to be used to record variations in neural activity when patients receive drug-related stimuli or experience the desire for drug taking.

When neurons change their activity patterns, such as during different phases of addiction, the local hemodynamic conditions in the brain change, resulting in neurovascular coupling (86). Whereas EEG can monitor electrical signals in the brain, fNIRS is a popular wearable device for monitoring the hemodynamic conditions of the brain, enabling these two to be used in combination. For example, the prefrontal, dorsolateral prefrontal, and orbitofrontal cortices are responsible for decision-making (30). When patients with drug addiction use the drug or receive cues related to it, this can change the activity patterns of these brain regions. This results in alternations in oxygenated and deoxygenated hemoglobin concentrations, which can be recorded using fNIRS at the corresponding cortices (87). Studies have shown that changes in these concentrations in patients with drug addictions are different from those in healthy controls (88). In addition, with its advantages of being light, compact, wearable, highly accessible, and user-friendly, fNIRS is becoming a popular tool to study the effects of exercise on drug addictions. For instance, fNIRS has been used to evaluate the effects of spin training and strength training on those with a METH addiction (89). Another study used fNIRS to evaluate the effects of dancing and exercise on aspects of meth addiction (90). As well as its applications in analysis of the hemodynamic variations influenced by exercise, fNIRS has been used to investigate the relative hemodynamic changes at different cortical regions in the brain (91). For example, a classification algorithm based on the fNIRS signals at various brain cortices was used to distinguish addictions to different drugs.

Neurovascular coupling indicates that electrical neuron signals are closely related to hemodynamic conditions. In neurovascular coupling, when neuronal activity is elevated, more oxygen is delivered to the activated brain regions, resulting in a local increase in oxygenated hemoglobin. As EEG measures neural electrical activity whereas fNIRS monitors hemodynamic activity, multimodal EEG-fNIRS recording provides a more holistic measurement, enabling a more comprehensive understanding of the effects of drug use and withdrawal on the brain (86). This dual approach has recently been used in several studies, for example, multimodal EEG-fNIRS has been used to study the brain activity of those with a METH addiction under visual stimulation (92, 93), whereas another study used multimodal EEG-fNIRS for those with an opioid addiction (94).

A further benefit of wearable systems is that they can be applied without the limitations of time and location, with a minimal influence on social activity. Consequently, multimodal EEG-fNIRS systems are suitable for evaluation of the efficacy of treatment and rehabilitation. An important goal during treatment and rehabilitation is the reduction of the incidence of relapse. Craving is a key symptom that promotes relapse to drug use; thus, identifying brain activity patterns during, immediately before, and immediately following cravings could help to optimize treatment and rehabilitation programs. For example, if we could identify the neuronal signatures of craving, we could trigger a closed-loop stimulation protocol to combat these activity patterns, or provide alternative interventions. In addition, brain signal monitoring can provide more information on changes in psychological and physiological conditions than the wearables measuring ECG, HR, and GSR mentioned in section “3.2. Conventional detection techniques and emerging wearable techniques.” Notably, including as many modalities as possible in the wearables would increase their diagnostic precision.

It has been suggested that wearables could be used to track the efficacy of treatment of those with a drug addiction (65). However, the propensity for interventions based on this approach alone is limited. To more effectively reduce rates of drug use and relapse, a closed-loop system to provide real-time treatment that is customizable to each specific case is needed (95). Such a closed-loop system would consist of three parts. The first part is neuroimaging, which can be achieved using multimodal EEG-fNIRS devices. The second involves computational algorithms to identify the neuronal and/or hemodynamic activity biomarkers of addiction. Finally, the loop is closed by neuromodulation approaches that provide stimulation of targeted brain areas to combat pathological activity changes related to addiction.

## 4. Biomarkers of neuroimaging techniques

In a closed-loop system, the treatment protocol can be optimized according to the real-time signals from the neuroimaging recording. Approaches to stimulate cues to further evaluate the level of desire for the drug are introduced in section “4.1. Stimulation cues.” The EEG and fNIRS biomarkers used to identify drug addiction are presented in section “4.2. Biomarkers of EEG and fNIRS in METH addiction.”

### 4.1. Stimulation cues

Approaches to investigating brain signal changes influenced by drug addiction include comparing recorded signals from those suffering from addictions with control populations, or comparing the signals of those suffering from an addiction across the addiction cycle. One approach to minimize the acute and long-lasting effects of drug intake on the participants during attempts to identify brain signal biomarkers, drug-related cues can be applied as alternatives to drugs. This approach is used because the variations in autonomic nervous system and brain signals occur not only after the presentation of drug use but also after cues. To identify useful biomarkers to distinguish addiction and control groups, protocols including various stimulations to provoke cue reactivity have been proposed (96). Cues can be videos, photos, audios, or VR of the drugs or individuals using the drugs. This approach has been done before using MRI and EEG. In these studies, it was found that VR induced more cravings than other stimulation types (97, 98). Natural cues are included as important controls to identify drug-specific cue responses (99). Natural cues can be categorized into those with limited connections to drugs and those that share some features with drug-related cues. The former types may include natural scenes such as trees and flowers, while the latter types could be, for example, a person holding a screwdriver close to the face. This may trigger thoughts of METH use because a screwdriver may appear similar to the use of a long tube typically used for METH consumption. Therefore, placing a different tool of a similar shape close to the face can induce desire for the drug. Another example of the latter type is a light bulb, which may look similar to a METH pipe. It has been demonstrated that these natural cues have common features with the drug-related ones that in turn induce higher levels of craving and desire (100).

As drug-paired cues are highly salient, emotional responses are influenced when an individual receives cues (101). Therefore, when neuroimaging signals are recorded, scales are applied to evaluate changes in emotions, such as valence, arousal, and craving (100). Measuring both the neural signals and the score on emotional scales provide more comprehensive information for over multiple physiological and behavioral dimensions.

Although cues provide an opportunity to distinguish those suffering from an addiction from healthy controls, experiments in which drug-related cues are provided to the participants have potential ethical issues. For example, the risk of relapse may increase after stimulation by drug-related cues. One alternative approach has been recorded, brain signals when participants are involved in cognitive tasks, to differentiate those suffering from an addiction from healthy controls (32); for example, in one study, EEG signals were analyzed when patients with METH addiction performed cognitive tasks, including the N-back task to assess working memory and Stroop task to assess attention (102).

Furthermore, it has been reported that without cue stimulation and cognitive testing, the resting state brain of those suffering from an addiction and healthy individuals exhibit differences (94, 103). Monitoring the brain signals altered by the METH addiction and tracking the recovery process at resting state is an attractive approach because no additional efforts are needed to implement the drug-related cues and synchronize the cues and recorded signals or to arrange a supervisor to conduct cognitive assessments.

### 4.2. Biomarkers of EEG and fNIRS in METH addiction

Biomarkers of neuroimaging techniques are not only used to distinguish those suffering from drug addiction from healthy controls, but they are also widely used to evaluate the efficacy of abstinence, exercise, and medical interventions. The biomarkers of EEG signals that have been shown to characterize the brain activity of those suffering from METH addiction are listed in Tables 1, 2. The biomarkers were determined by comparing the recorded EEG signals from patients with a METH addiction with those of healthy controls. The brain signals were recorded when participants received various METH-related cues (see section “4.1. Stimulation cues”), after conducting cognitive tasks, or during resting states. The EEG biomarkers that can identify the patients with a METH addiction can be categorized into three types based on the analysis approaches used. First, the time-domain EEG signals can be converted to frequency-domain signals to reveal the spectral information of EEG sub-bands. The sub-bands of each frequency range represent different conditions affected by METH (54, 85, 104–110) of Table 1. The entropy of the EEG signals at a specific frequency range can be derived from the spectrum of that frequency range (104). The second type of biomarkers are based on time-domain signals. As EEG records neural activity on a millisecond timescale, the neural signals triggered by stimuli (visual, audio, etc.) show specific wave forms, namely the event-related potential (ERP) (111, 112).

**TABLE 1:** Biomarkers of EEG signals in frequency and time domains on patients with methamphetamine addiction.

References	Comparison conditions	Groups for comparison	Number of the electrodes and their locations	Biomarkers	Main limitations
Newton et al. (104)	Eye-closed resting state during abstinence	METH (with 4 days of abstinence) versus HC	35 electrodes distributed across the scalp	Increases: delta and theta bands across the scalp	Patients with another period of abstinence can be investigated.
Newton et al. (105)	Eye-closed and cognitive tasks	METH (with 4 days of abstinence) versus HC	35 electrodes distributed across the scalp	Increases: theta band increases with the increasing of the reaction time of cognitive tasks	Difficult to identify that the EEG biomarkers have resulted from METH use disorder or other health issues.
Yun et al. (106)	METH users at abstinence stage. Eye-closed resting state.	METH versus HC	16 electrodes distributed across the scalp	Decreases: approximate entropy	Patients are separated in to high- and low-dose of METH groups by their duration of METH use, not by the cumulated dose.
Kalechstein et al. (107)	2.5 h of neurocognitive assessment tests	METH versus HC	35 electrodes distributed across the scalp	Increases: theta band correlation with poor performances on cognitive tasks	The changes of biomarkers along with varies abstinence time can be further studied.
Howells et al. (108)	Resting eyes closed, eyes-open and a cognitive task	METH versus HC	6 channels (F3, F4, C3, C4, P4, and P4)	Increases: delta/alpha ratio	Future studies are needed, including a wider variety of mental disorders in METH patients.
Ding et al. (109)	Drug-related and neutral VR	(1) METH versus HC (2) METH versus neutral cues	5 channels (Fpz, AF7, AF8, TP9, and TP10)	(1) Increases: beta and gamma Decrease: delta and alpha (2) Decrease: delta, theta, and alpha	Be cautious when applying the machine learning modal built from male-only patients on female patients.
Lu et al. (85)	METH users received anaerobic resistance treatment (RT) and aerobic cycling treatment (CT). Test conditions are eyes closed (EC), eyes open (EO), and drug-related and neutral cues	(1) METH with exercise (RT or CT) versus METH without any exercise (2) Before and after exercises	64 electrodes distributed across the scalp	(1) Increases: absolute power of theta, alpha, and beta bands on RT group during EC; the alpha block rate on RT group during EO and drug cues Decreases: mean frequency on RT group during drug cues (2) Decreases: brain lateralization index on RT group during EC	Lack of a healthy control group.
Minnerly et al. (110)	Eye-closed resting state	METH versus HC	19 electrodes distributed across the scalp	Increases: delta and theta bands across scalp Decrease: alpha	Did not apply AI algorithm to reduce the analysis workload of extensive data.
Zhao et al. (54)	Visual stimuli (video) then eyes-closed resting state	METH users in abstinence for 1–3 months versus other abstinence lengths	128 electrodes distributed across the scalp	Increases: beta across scalp Decreases: theta and alpha	No comparison with healthy control and no longitudinal measurements on the same patient.
Shahmohammadi et al. (111)	METH users at abstinence stage. Visual stimuli (drug-related, drugs and neutral images).	METH versus HC	32 electrodes distributed across the scalp	Increases: P300 peaks of the event-related potentials (ERP)	All METH patients had history of cigarette smoking and no healthy subject had the history. This might influence to results.
Khajehpour et al. (112)	Visual stimuli (drugs and neutral images) after tDCS	Biomarkers mean the difference of the biomarkers of watching drug related cues and neutral cues. METE users before versus after treated with tDCS.	62 electrodes distributed across the scalp	Increases: P3-related late positive potential (LPP) component of the ERP Decrease: P3 component	Repetitive tDCS was not applied, only a single session tDCS is conducted.

GSR, galvanic skin response; HC, healthy control; tDCS, transcranial direct current stimulation; METH, methamphetamine.

**TABLE 2:** Biomarkers of EEG signals in functional connectivity (FC) network and network topological properties on patients with METH addiction.

References	Comparison conditions	Groups for comparison	Number of electrodes and their locations	Biomarkers*	Main limitations
Ahmadlou et al. (113)	Resting state	METH (with 1–3 weeks of abstinence) versus HC	31 channels distributed across the scalp	Increases: CC and the CC/L of gamma band in the small world network (SWN) Decreases: L of the gamma band in the SWN	The backgrounds of the METH and HC groups may not be similar.
Khajehpour et al. (114)	Resting state	METH (during 1–6 months of abstinence) versus HC	62 electrodes distributed across the scalp	Increases: CC and SWI in delta and gamma frequency bands Decreases: L in delta and gamma frequency bands Abnormal changes: inter-regional connectivity and network hubs in all the frequency bands	HC can have a smoking, drinking, or caffeine history, which may affect the results.
Khajehpour et al. (115)	Resting state	METH versus HC	64 channels on the overall scalp	Decreases: WPLI of beta bends	Only male patients were included.
Shafiee-Kandjani et al. (116)	Resting eyes closed and eyes open	METH versus HC	19 channels on occipital, temporal, frontal, and parietal lobes	Decreases: coherences of the delta and theta band on the left frontoparietal cortices (F3Fz and C3Cz)	Coherences were used to study the linear relationship of the signals. However, brain signals seem to have more non-linearity properties.
Zhao et al. (54)	Visual stimuli (video) then eyes-closed resting state	METH users abstinent for 1–3 months versus other abstinence lengths	128 channels distributed across the scalp	Increases: WPLI between medial prefrontal cortex and bilateral orbital gyrus in the beta band	No comparison with healthy control and no longitudinal measurements on the same patient.
Qi et al. (117)	Resting state with eyes open. METH users in control group, dancing group, and bicycling group	METH with exercises versus control group	64 channels distributed across the scalp	Increase: brain flexibility and network connectivity entropy Decrease: mean frequency and beta relative power	Lack of data from healthy subjects.
Chen et al. (102)	Resting state	METH versus HC	64 channels distributed across the scalp	Increases: GEV of 1 microstate (customized microstate C) Decreases: MMD of 2 microstates (customized microstates A and B); GEV of 1 microstate (microstate B)	Simultaneously MRI recording will be helpful to compare with the microstates data.
Lin et al. (118)	Resting state with eyes open; then visual stimuli of METH cues with VR	(1) METH under cues versus resting; (2) METH versus HC	32 channels on the overall scalp	(1) Increases: coverage and occurrence of microstate B, transitions of microstates B → D and D→ B pairs Decreases: coverage, duration, and occurrence of microstate A, occurrence of microstate C, transitions of microstates A → C and C → A pairs (2) Increases: coverage of microstate A Decreases: coverage and occurrence of microstate B	The number of microstates was limited to 4 during the analysis. Results might change with other numbers of microstates.

CC, clustering coefficient; GEV, global explained variance; HC, healthy control; L, characteristic path length; METH, methamphetamine; MMD, mean microstate duration; SWI, small-world index; WPLI, weighted phase lag index. *The microstates discussed in this table were derived based on individual EEG recordings (102, 118). In other words, microstates with the same names may have different topographies in different studies.

The third type of EEG biomarker can be visualized by the topography of the data (Table 2). The advantage of plotting brain signals topographically is that signal variations throughout the cortex can be evaluated. Brain activity is typically not localized to one specific region, but rather observed as coordinated activity throughout multiple connected regions. Functional connectivity (FC) in this case refers to the level of connectivity of each channel and the connectivity between cortical regions (54, 113–117). FC can be analyzed by processing an EEG signal using various approaches. Some studies have considered the coherence of the EEG sub-bands of electrode pairs (113, 116). However, coherence neglects the non-linear relationship between the channels. Therefore, another study used visibility graph similarity as a non-linear approach (113). Later, the weighted phase lag index (WPLI), a modification of the phase lag index (PLI), was used for FC analysis. Compared with coherence, PLI (117) and WPLI can better indicate the delays in signals between channels (54, 114, 115). Parameters of such graph theory analyses include node strength, characteristic path length (L), clustering coefficient (CC), and small-world index (SWI), which is derived as the ratio of CC to L. Moreover, the network hub(s) can be identified by the node strength, betweenness centrality, and eigenvalue centrality. These parameters can be used as features of ML models to classify and differentiate those with METH addiction from healthy subjects (115).

Other EEG biomarkers represented by topography include EEG microstates that show the spatial distribution of electrical signals recorded by the electrodes over the scalp (102, 118). The dynamic changes of these electric potential states can be validated by examining a consecutive set of topographies. EEG microstates represent an emerging technique for analysis of EEG signals, especially at resting states. The parameters used to investigate microstates are the mean states duration, total time covered/coverage ratio, global explained variance (GEV), occurrence, and transition probability. Studies to date have been limited because the number and type of microstates are optimized differently, depending on the database used. Fortunately, most microstates derived from the resting state of healthy subjects of different studies comprise four representative states, where each state corresponds to specific neural activity patterns, such as visual or audio responses. However, microstates of people with neurological diseases or actively engaged in task-related states (e.g., receiving cues or being involved in a cognitive task) vary across studies (118). Therefore, interpretation of results is not always straightforward, and comparisons between different studies can be challenging.

In the EEG spectrum analyses summarized in Table 1, one approach is that an individual electrode is inspected only when the number of electrodes is small (108). Alternatively, the spectrum of individual electrodes is calculated, then representative channels are selected for further investigation (111). In studies with larger numbers of electrodes, the average EEG spectrum of all electrodes is often investigated (54, 85, 104–107, 109, 112). Lu et al. and Minnerly et al. studied changes in the EEG spectrum of different brain regions (85, 110). The former separated the brain into four areas, whereas the latter separated the cortices using five different approaches. A lower number of EEG channels reduces the preparation time when the region of interest is well known. However, increasing the number of electrodes allows for studying FC across various brain regions.

Some studies had explored EEG biomarkers when subjects had their eyes closed but were not asleep; this is done to reduce the disturbance due to non-task related visual stimuli (104–106, 108, 110). Other studies report the identification of biomarkers specifically when the subjects had received cues (85, 109, 111, 112). Some studies included cognitive tasks in the experiment protocols. However, most of these studies only analyzed the correlation of the level of cognitive impairment (e.g., the reaction time and the response accuracy) and the EEG spectrum (105, 107). Few studies have monitored the variation in the EEG spectrum during cognitive tasks (108). For future applications in closed-loop neuromodulation systems, the biomarkers found when the participants were simultaneously receiving cues may be more helpful than, e.g., at a resting state, as the use of cues can more accurately simulate the conditions of having a desire for a drug.

In addition to the EEG signal, FC is often studied in the resting state as well. In the task state, the connectivity needs to be analyzed in every pair of channels at every point of interest, resulting in a heavy computational load. This is because the brain is engaged in various tasks at different stages along with the task. Only the data of a selected resting time period is calculated in the resting state. For this reason, in the task state, only the channels of interest are often analyzed to reduce computational load (54). Only one study has evaluated the effects of exercise on those with METH addiction using EEG signals (116). In this study, the EEG signals were not recorded simultaneously during the exercise, but rather before and after the acute and long-term period of exercise.

An fNIRS device is easy to wear without time-consuming preparation such as is required for EEG gel electrodes. Therefore, fNIRS devices have been widely used to study the effects of exercise on patients with METH addiction during abstinence. Optodes of fNIRS are often mounted on the prefrontal cortex and the motor cortex to study the influence of METH on decision-making as well as on cognition and motion abilities. The biomarkers of fNIRS signals are listed in Table 3. These biomarkers include a variation of hemoglobin concentration (34, 87, 89, 90, 119–122) and parameters of functional connectivity (34, 88, 121, 122). The most used hemodynamic parameter is the concentration variation of oxygenated hemoglobin, △[OxyHb]. Deoxygenated hemoglobin concentration variation and regional cerebral oxygen saturation were not found to have been used in METH-related applications. To calculate the FC of channel pairs, Pearson’s correlation is the most frequently used approach (34, 121, 122). Some studies have used the coherence of the channel pairs (88, 89). In this case, the fNIRS signals must first be converted to various frequency ranges. One study calculated the coherence of four frequency ranges (89), whereas another used only one (88). Optimization of the frequency of coherence evaluation needs further study. The fNIRS parameters include global efficiency and local efficiency, in addition to the graph theory parameters of path length and clustering coefficients (122).

**TABLE 3:** Biomarkers of fNIRS signals recorded on patients with methamphetamine addiction. The biomarkers include variation of hemoglobin concentration and functional connectivity (FC).

References	Comparison conditions	Groups for comparison	Locations* and number of the channels	Biomarkers	Main limitations
Bu et al. (89)	METH users during resting and exercise: spinning training and strength training	(1) After exercises versus resting; (2) Strength versus spin training	Prefrontal cortex: 8, motor cortex: 16	(1) Increase: △[OxyHb], wavelet phase coherence (WPCO) at frequency intervals II and IV^†^ Decrease: WPCO at frequency interval I (2) Increase: △[OxyHb], WPCO at frequency intervals III and IV Decrease: WPCO at frequency intervals II	Longitudinal recordings can be carried out to explore the changes in the biomarkers.
Bu et al. (88)	METH users during resting and exercise: kick boxing	(1) METH group versus HC at resting and training states; (2) METH group during training versus resting	Prefrontal cortex: 8, motor cortex: 16	(1) Decrease: effective connectivity (EC) of some pair of channels (2) Decrease: EC of some pair of channels	Only signals when eyes closed were analyzed.
Wang et al. (119)	METH users exercising; then visual stimuli of images with food	After exercises versus control group (no exercise) when receiving cues with high-calorie food	OFC: 4, VLFPC: 4, DLFPC: 7, PPA: 5	Increase: △[OxyHb] of some channels at OFC	No measurements on healthy subjects for comparison.
Zhou et al. (90)	METH users exercising: dancing or treadmill; then visual stimuli of images with food	After versus before treadmill training when receiving cues with high-calorie food	OFC: 4, VLFPC: 4, DLFPC: 7, PPA: 5	Decrease: △[OxyHb] of one channel at left DLFPC	fNIRS cannot provide hemodynamic information in the deep brain.
Tao et al. (120)	METH users exercising: dancing or cycling, then visual stimuli of images which caused negative emotions	After versus before dancing, when receiving cues with negative images	OFC: 4, VLFPC: 4, DLFPC: 7, PPA: 5	Decrease: △[OxyHb] of one channel at DLFPC	No measurements on healthy subjects for comparison.
Gao et al. (121)	METH users exercising: cycle ergometer with moderate or high intensity	METH with high intensity versus moderate intensity of exercises	OFC: 4, VLFPC: 4, DLFPC: 7, PPA: 5	Increase: △[OxyHb] at PFC and DLFPC, FC of left DLPFC and OFC	No measurements on healthy subjects for comparison.
Qi et al. (34)	METH users exercising: VR cycling, then visual stimuli of images with drug-related and neutral cues	(1) Drug-related versus neutral cues; (2) After versus before exercise when seeing drug-related cues	DLPFC: 8, VLPFC: 8, PM and SMA: 6, M1: 4 S1: 6, FPA: 2, OFC: 4, FEF: 4	(1) Increase: △[OxyHb] at OFC and DLPFC; (2) Increase: FC between PFC and motor cortex, between VLPFC and other cortices; Decreases: △[OxyHb] at OFC and DLPFC	No measurements on healthy subjects for comparison.
Qi et al. (122)	METH users exercising: VR cycling. Before and after the exercise session, a cognitive task (Stroop task) was carried out	(1) After versus before exercise during cognitive task; (2) After versus before exercise during resting	DLPFC: 8, VLPFC: 8, PM and SMA: 6, M1: 4 S1: 6, FPA: 2, OFC: 4, FEF: 4	(1) Increase: • Cortical activation at DLPFC; • C_p_, E_local_, E_global_, and E_nodal_^‡^; 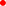 Decrease: L_p_ (2) FC between prefrontal cortex and motor cortex	No measurements on healthy subjects for comparison.
Gu et al. (87)	Patients with various drug addiction drug-related cues	METH group versus heroin group and mixed group	DLPFC: 16, VLPFC: 6, FPA: 16, OFC: 10	Increase: activation of OFC	Limited number of subjects.

FC, functional connectivity; Δ[OxyHb], concentration variation of oxygenated hemoglobin. *DLPFC, dorsolateral prefrontal cortex; FEF, frontal eye field; FPA, frontopolar cortex; M1, primary motor cortex; OFC, orbitofrontal cortex; PM, pre-motor cortex; S1, primary somatosensory cortex; SMA, supplementary motor cortex; VLPFC, ventrolateral prefrontal cortex. ^†^Frequency intervals of fNIRS signals: interval I: 0.6–2 Hz, interval II: 0.145–0.6 Hz, interval III: 0.052–0.145 Hz, and interval IV: 0.021–0.052 Hz. ^‡^Network efficiency metrics of the small world properties: clustering coefficient (C_p_), characteristic path length (L_p_), nodal efficiency (E_nodal_), network global efficiency (E_global_), and local efficiency (E_local_).

The fNIRS studies in individual with METH addiction were all conducted in the past 3–4 years. Interestingly, all these studies were carried out in China, specifically by the groups of Dong (34, 88, 89, 122), Chen, and Zhou (90, 119, 121). This might be because of an increasing focus in mainland China on treating drug addiction *via* scientific approaches that can quantify the efficacy in treatment outcomes (123).

Most of the experimental groups in the studies summarized in Tables 1–3 consisted of those with a METH addiction before or during abstinence. This was likely because rehabilitation centers are the most convenient places to recruit participants for those studies. However, limited studies have reported how long that those in recovery from METH addiction had been abstinent, and the amount of METH taken has been seldom reported in a systematic fashion. It has been demonstrated that brain activity changes with abstinence duration (54), and that the propensity for relapse varies as a function of abstinence duration. Thus, more detailed research on a wider variety of METH user groups using fNIRS needs to be performed, and long-term follow-up of users after withdrawal may yield important insights toward development more efficacious treatments.

## 5. Neuromodulation treatments for METH addiction

**FIGURE 2:**
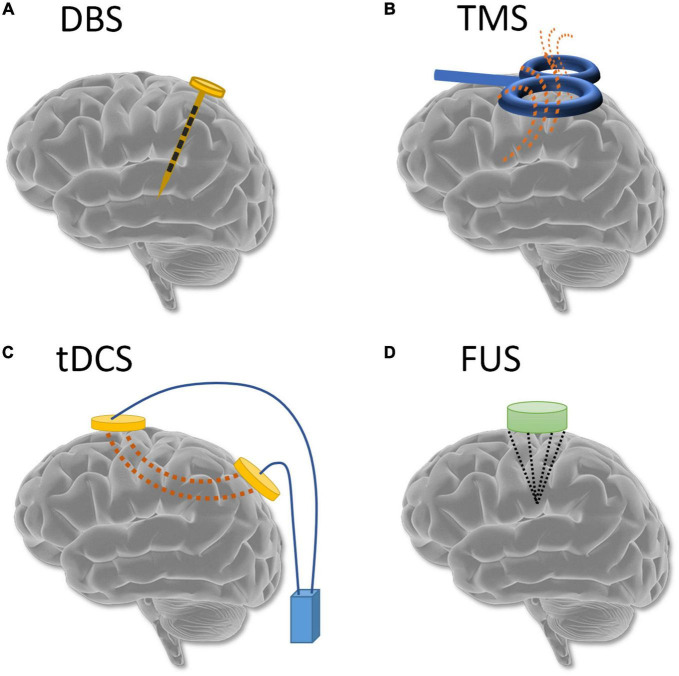
Common neuromodulation techniques: **(A)** deep brain stimulation; **(B)** transcranial magnetic stimulation; **(C)** transcranial direct current stimulation; and **(D)** transcranial ultrasound stimulation.

Advances in neurophysiology together with the neuroimaging technologies discussed here have led to the identification of some mechanisms underlying METH addiction disorders. Many studies have suggested that impaired self-control, irritability, compulsive consumption, etc., are caused by dysregulation and malfunction of specific brain circuits. Traditional pharmacotherapy, one of the most commonly applied interventions, can be viewed as a type of neural circuit modulation. However, traditional interventions lack spatial and temporal specificity of action. Neuromodulation, a novel approach that can modulate brain activity with spatiotemporal precision, has shown efficacy and is a promising treatment for addiction disorders (38) (Figure 2). Invasive neuromodulation techniques such as DBS, vagus nerve stimulation, etc., require surgery to implant a device and are usually used in severe and otherwise intractable brain disorders such as Alzheimer’s disease and epilepsy (124), with some promising results. However, the safety and long-term biocompatibility are still challenging issues to be overcome for invasive neuromodulation. In contrast, non-invasive techniques such as TMS, tDCS, and transcranial ultrasound stimulation are widely used as research tools to probe affected circuits and also as therapeutic interventions for a variety of neurological and psychiatric disorders, with encouraging results (125). In the remainder of this section, we focus on non-invasive neuromodulation devices for treatment of METH addiction.

### 5.1. tDCS for methamphetamine addiction

Transcranial direct current stimulation uses a constant low-intensity current that passes through two electrodes attached to the scalp of the participant to modulate neural activity. During tDCS modulation, a current flows between the electrodes and passes through the brain. A positive anodal current is generally considered to depolarize the neurons, thereby increasing cortical excitability and behaviors associated with the cortical region under the electrode. On the other hand, a negative cathodal current hyperpolarizes neurons, thereby inhibiting action potentials and behaviors in the corresponding cortical region.

A standard apparatus for tDCS stimulation, as shown in Figure 2C, includes a target electrode, used to stimulate the region of interest as determined by the modulation task. A reference electrode is commonly placed opposite the target electrode. Modeling studies have shown that the shorter the distance between the two electrodes, the more susceptible the current is to shunting effects. Generally, large distances between the scalp electrodes are expected to increase cortical modulation, allowing the current to be drawn through the cortex rather than shunted across the scalp (126). As the advisable safety threshold for human studies is 2 mA (127), most tDCS stimulation studies use currents between 0.5 and 2 mA with a duration between 5 and 30 min.

The dorsolateral prefrontal cortex (DLPFC) is the area most selected for tDCS stimulation of subjects with METH addiction, as dysfunction in this area has been reported frequently among these individuals. Moreover, DLPFC can also be easily targeted in a non-invasive fashion. Table 4 summarizes recent studies that have used tDCS for the treatment of addiction. Shahbabaie et al. conducted three 20-min sessions with 30 participants in a double-blinded sham-controlled trial (128). They found that the anodal tDCS of the right DLPFC decreased immediate craving at rest; however, cue-induced METH cravings could increase under active online stimulation of the right DLPFC. In another study, tDCS experiments lasting for half a year were conducted. After 20 sessions (1 month), the subject reported a significant reduction in craving and was able to control their cravings. Four booster tDCS sessions were given in the following 5 months as symptom-triggered therapy (129). These booster tDCS treatments were shown to be helpful in controlling psychological stress and drug cravings. Shahbabaie et al. conducted a double-blinded sham-controlled crossover study with 15 males with METH addiction (130). For each anticipant, 20 min sessions of real or sham 2-mA tDCS were applied over the DLPFC on two separate days in a random order. Participants receiving the real tDCS stimulation showed significant decreases in cravings. However, another clinical trial reported that cue-induced craving was reduced significantly but there were no significant effects on spontaneous cravings (131). More recently, a randomized controlled trial investigated the effects of tDCS on cue-induced craving; consistent with the findings of other studies, the results showed that tDCS could significantly reduce cravings (132). Jiang et al. applied tDCS and used two-choice oddball tasks to evaluate behavioral impulsivity prior to and after the treatment. However, they found that their protocol was not optimized to reduce symptoms associated with METH addiction (42). Khajehpour et al. conducted a sham-controlled tDCS stimulation experiment with 42 male participants with METH addiction (112). The results showed that tDCS could mitigate initial attention bias to METH stimuli. Overall, the findings summarized in Table 4 indicate that tDCS stimulation of DLPFC likely can play an active role in suppressing cravings.

**TABLE 4:** Examples of tDCS treatments in patients with methamphetamine addiction.

References	Number of subjects METH patients	Treatment sessions	Stimulation parameters (current, duration, and location)	Effects	Main limitations
Shahbabaie et al. (128)	30 males	3 sessions. At least 72 h between two sessions.	2 mA, 20 min. Anode: F4 (right); cathode: contralateral supraorbital area.	Reduced craving at resting state. Increased craving during meth-related cue exposure.	The effects might be transient. Long-term effects need to be explored.
Shariatirad et al. (129)	1 male	5 sessions a week, for 4 weeks. During 6-month follow-up, booster tDCS on days 67, 70, 72, and 88.	2 mA, 20 min. Anode: right DLPFC; cathode: over right arm.	Reduced drug cravings as measured by DDQ and LDQ.	This is a case report.
Shahbabaie et al. (130)	15 males	Two separate days, one-week washout period.	2 mA, 20 min. Anode: F4 (right); cathode: F3 (left).	Significant decrease of craving after tDCS, modulation of DMN, ECN, and SN.	A limited number of subjects.
Anaraki et al. (131)	30 males	5 sessions.	2 mA, 20 min. Anode: F4 (right); cathode: F3 (left).	Cue-induced cravings reduced significantly, no significant change in instant cravings.	Lack of longitudinal recordings to analyze the long-term effect of tDCS.
Xu et al. (132)	75 females	CCAT + tDCS, 5 sessions per week, for 4 weeks.	1.5 mA, 20 min. Anode: F4 (right); cathode: F3 (left).	Reduced cue-induced cravings.	Lack of longitudinal recordings to analyze the long-term effect of tDCS.
Jiang et al. (42)	45 males	5 days daily.	2 mA, 20 min. Anodal: F4 (right); cathode: F3 (left).	Counterproductively increased impulsivity.	No simultaneous neuroimaging signals to provide the real-time effect of tDCS.
Khajehpour et al. (112)	42 males	1	2 mA, 20 min. Anode: F4 (right); cathode: F3 (left).	Mitigated initial attention bias but not sustained motivated attention to METH related stimuli.	Repetitive tDCS was not applied. Only a single-session tDCS is conducted.

CCAT, computerized cognitive addiction therapy; DDQ, Desire for Drug Questionnaire; DLPFC, dorsolateral prefrontal cortex; DMN, default mode network; ECN, executive control network; LDQ, Leeds Dependence Questionnaire; METH patients, patients who were addicted to methamphetamine; SN, salience network.

The most common electrode locations in these studies are F4 (right DLPFC) for the anodal and F3 (left DLPFC) for the cathode electrodes. In these other studies, only two of the studies listed in Table 4 placed the cathode at other locations. One placed the cathode electrode on the left supraorbital area (128), and the other placed it over the right arm (129). Regarding the current used for stimulation, most studies used 2 mA, while one used 1.5 mA (132). For the evaluation of treatment effects, Shahbabaie et al. used EEG, Khajehpour et al. used fMRI, and the others used VAS or performance on cognitive tasks.

### 5.2. TMS for methamphetamine addiction

Transcranial magnetic stimulation techniques use a strong electrical current through an electromagnetic coil to generate magnetic pulses (Figure 2B). The magnetic pulse-induced electrical activity in the targeted brain area serves the purpose of neuromodulation. In practice, rTMS is used to elicit neuromodulation and neuroplasticity. Unlike tDCS, where the excitation and inhibition are controlled by anodal and cathodal stimulations, respectively, in rTMS the frequency drives the direction of neuromodulation (133). Frequencies over 5 Hz have been shown to increase cortical excitability, whereas low frequency rTMS, such as 1 Hz, decreases cortical excitability. Still another type of rTMS is the theta burst stimulation (TBS, a variation of high frequency rTMS) has also been applied to evaluate the effects on withdrawal of METH (134–137). TBS can be further separated into continuous TBS (cTBS) and intermittent TBS (iTBS), depending on the existence of intertrain intervals. The TBS consists of 3 pulses at 50 Hz forming 1 burst, and the bursts are repeated at 5 Hz. In cTBS the bursts are applied continuously while in iTBS, the bursts are applied with 2 s on and 8 s off, continuing for a designed number of cycles. TMS treatments have been reported to not only reduce feelings of craving in patients with METH addiction, but also to reduce negative emotions (such as depression and anxiety) and improve cognitive function (138–140). TMS stimulation on DLPFC also an FDA-approved treatment for depression (FDA approval K061053). Therefore, like tDCS, DLPFC is also the most commonly targeted stimulation area to modulate METH addiction (Table 5). A pilot study by Li et al. reported that low-frequency rTMS increased cravings of METH patients receiving drug-related cues (141). Liang et al. developed a protocol with 2 days rest between two 5-day treatments (142). Chen et al. designed a TMS protocol to stimulate not only the DLPFC but also the ventromedial prefrontal cortex (vmPFC) and reported an improved effect of this combined protocol (125). Zhao et al. showed that high-frequency rTMS over the DLPFC and low-frequency rTMS over the right DLPFC could reduce cravings for METH (135). Other studies have shown that TMS of the left DLPFC is effective to reduce METH addiction (142, 143). Wen et al. reported a decrease in theta/beta ratio after participants had received TMS treatment (144). Liu et al. showed that iTBS, which is a more time-efficient protocol, had similar effects to those of rTMS at 10 Hz (145). Besides the studies discussed in Table 5, some ongoing protocols have been proposed to investigate the effects of TMS treatment on METH addiction. One protocol proposes to analyze the power spectrum of EEG sub-bands when individuals receive METH-related VR cues before and after the cTBS treatment (137). Another protocol is designed to examine the effects of various stimulation parameters (146). Protocols are not listed in Table 5 since no experimental data are reported yet.

**TABLE 5:** Examples of TMS treatments for methamphetamine addiction.

References	Groups for comparison	Treatment sessions	Brain area, coil type, and stimulation parameters (frequency, intensity, total number of pulses, and duration of treatment)	Effects	Main limitation
Li et al. (141)	10 METH* versus 8 HC. Real versus sham TMS.	One session: 15 min of sham and real TMS separated by 1 h.	Left DLPFC, figure-of-eight, 1 Hz rTMS, 100% rMT, 900 pulses, 15 min.	Increase: cue-induced craving in METH.	The first studies to explore the TMS effect on METH addiction. Many stimulation parameters can be further optimized.
Liang et al. (142)	50 males (1–15 days of abstinence). Real versus sham TMS.	5 days treatments, then 2 days of rest, followed by another 5 days of treatments.	Left DLPFC, 10 Hz rTMS (5 s on and 10 s off), 100% rMT, 2,000 pulses, 10 min.	Decrease: craving and withdrawal symptoms.	The long-term effect of rTMS needs to be further explored.
Chen et al. (125)	74 METH, separated into 3 real (A, B, and C) and 1 sham TMS.	One session/day and 5 days/week, in total 10 sessions over 2 weeks.	(A) Left DLPFC, figure-of-eight, 2 s on and 8 s off iTBS, 100% rMT, 900 pulses, 5 min; (B) Left vmPFC, butterfly coil, 900 pulses cTBS, 110% rMT, 60 s; (C) A combination of the above two protocols.	Decrease: cue-induced craving for all three groups. Group 3 was most effective.	The long-term effect of treatment needs to be further explored.
Zhao et al. (135)	83 METH, separated into 3 TMS groups (A, B, and C)	Twice daily over 5 days for a total of 10 sessions.	(A) Left DLPFC, figure-of-eight, 2 s on and 8 s off iTBS, 70% rMT, 600 pulses, 3 min; (B) Right DLPFC, round-shaped, 600 pulses cTBS, 70% rMT, 40 s; (C) Left DLPFC, figure-of-eight, 600 pulses cTBS, 70% rMT, 40 s.	Decrease: cue-induced cravings for groups (A) and (B).	The long-term effect of treatment needs to be further explored.
Wang et al. (143)	66 METH (within 3 months of detoxification). Real and sham TMS.	5 days/week, 20 sessions.	Left DLPFC, figure-of-eight, 2 s on and 8 s off iTBS, 100% rMT, 600 pulses, 3 min.	Decrease: cue-induced cravings.	The long-term effect of treatment needs to be further explored.
Liu et al. (145)	20 male METH, separated into 2 TMS groups.	First 10 days daily, then on days 15 and 20.	(A) Left DLPFC, circular, 10 Hz rTMS (5 s on and 10 s off), 100% rMT, 2,000 pulses, 10 min; (B) Left DLPFC, 2 s on and 8 s off iTBS, 100% rMT, 600 pulses, 190 s.	Decrease: cue-induced craving for both groups.	iTBS had a much shorter stimulation time compared to rTMS, which might affect the results.
Wen et al. (144)	15 female METH. Real versus sham TMS.	Two separate sessions within 1 week	Left DLPFC, figure-of-eight, 2 s on and 8 s off iTBS, 80% rMT, 1,800 pulses, 10 min.	Decrease: frontal EEG theta/beta ratio during cue-related VR scenes.	The long-term effect of treatment needs to be further explored.

cTBS, continuous theta burst stimulation; HC, healthy control; iTBS, intermittent theta burst stimulation; METH, individuals with methamphetamine addiction; rMT, resting motor threshold; vmPFC, ventromedial prefrontal cortex; VR, virtual reality. *Individuals with current methamphetamine dependence and non-treatment seeking.

**FIGURE 3:**
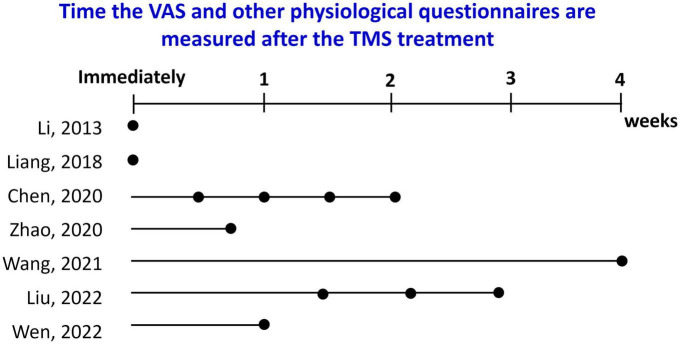
The time when the VAS and other physiological questionnaires (if available) were measured after TMS treatment for the studies is listed in Table 5.

All studies in Table 5 apply self-rated VAS scores to assess the effects of TMS. Only one study uses EEG signals together with VAS scores to assess the effects (144). Some studies use images (125, 141, 143, 145), some use videos (135) and still others use VR as the cues (144). One study asks the participants to actively interact with the tools used for METH consumption (142). Since the FDA has approved TMS to treat depression, effects on emotions are compared before and after the treatments. The most common assessment is self-rating questionnaires regarding depression, anxiety, and withdrawal symptom. Results have indicated that the VAS, depression, anxiety, and withdrawal symptoms all decrease after TMS treatment (145). iTBS has become a more popular treatment approach because it takes less time to achieve the same therapeutic effect as rTMS (145). However, comparing the long-term effects of various protocols is challenging because the results reported after TMS treatments are not over the same time frame. For example, the VAS scores are measured either immediately or 4 weeks after the TMS (Figure 3). In addition, stimulation intensity is often non-consistent across studies: the published intensity has varied from 70% resting motor threshold (rMT) to 110% rMT. In the future, more data regarding the stimulation intensities and long-term effects will be needed.

Another potential confound of the studies being discussed is that the patients population within a given study is either all male, or all female. This is because of a single-gender policy that applies at the rehabilitation centers. With the wearable closed-loop system we propose in the following section, assessing potential effects of TMS in both males and females would be easier to implement without these limitations.

## 6. Challenges and future trends in treatment of METH addiction

In section “4. Biomarkers of neuroimaging techniques,” we summarized the evidence that neuroimaging biomarkers can be used to distinguish patients with METH addiction from healthy individuals and evaluated the efficacy of various types of treatment, such as exercise training and neuromodulations. Furthermore, in section “5. Neuromodulation treatments for METH addiction,” we discussed the results of studies that have used TMS on the DLPFC to reduce cravings resulting from drug-related cues in METH user groups. However, these results were based on offline signal processing, which does not the variations of brain activity during treatment in real time, which is essential for closed-loop therapeutics. In addition, bulky TMS systems hinder wider applications to increase the efficacy of the therapy. We propose a wearable closed-loop neuromodulation to efficiently treat METH addiction (147).

### 6.1. The modules of the proposed wearable closed-loop neuromodulation system

**FIGURE 4:**
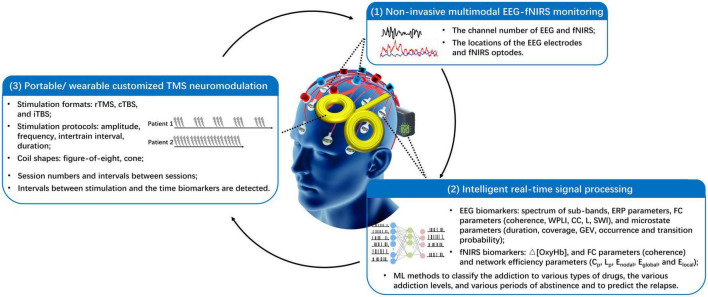
Proposed intelligent closed-loop TMS neuromodulation to treat methamphetamine addiction. It uses EEG-fNIRS measurements and is composed of three main parts: a real-time brain signal monitoring interface, an artificial intelligence signal processing block, and a customized neuromodulation system. C_p_ and CC, clustering coefficient; cTBS, continuous theta burst stimulation; E_global_, network global efficiency; E_local_, local efficiency; E_nodal_, nodal efficiency; ERP, event-related potential; FC, functional connectivity; GEV, global explained variance; iTBS, intermittent theta burst stimulation; L_p_ and L, characteristic path length; rTMS, repetitive TMS; ML, machine learning; Δ[OxyHb], concentration variation of oxygenated hemoglobin; SWI, small-world index; WPLI, weighted phase lag index.

This system consists of multimodal EEG-fNIRS combined with TMS (Figure 4), a combination that could potentially overcome the limitations of current detection and treatment approaches. The advantages of individual module in the proposed closed-loop system are described in this section. Furthermore, an example of the scenario of applying the closed-loop system is explained in section “6.2. Apply our closed-loop system to treat METH addiction.”

#### 6.1.1. Multimodal EEG-fNIRS neuroimaging

Limited studies have combined multimodal EEG and fNIRS for METH-related applications. Chen et al. used concurrent EEG-fNIRS monitoring to evaluate the influence of aerobic exercise on patients with METH addiction during cognitive tasks (35). In the studies presented in section “4.2. Biomarkers of EEG and fNIRS in METH addiction,” most fNIRS measurements carried out on patients with METH addiction focused on signals from frontal cortices, whereas they recorded EEG signals from several channels covering a larger area of the scalp. Recording fNIRS signals from cortical regions other than the frontal cortices could be interesting, as this would both enable analysis of multimodal signals of nearby EEG and fNIRS signals to study the effects of addiction on neurovascular coupling mechanisms, and could also increase the data from which to extract useful biomarkers to identify phases of addiction, withdrawal, and relapse in METH users. Many commercially available systems support multimodal EEG and fNIRS measurements simultaneously, and other multimodal EEG-fNIRS systems are under development because of research to achieve more compact and user-friendly systems.

#### 6.1.2. Analysis of recorded neural signals

To demonstrate the efficacy of TMS treatment, neural signal analyses are carried out offline. Thus, the alternations in brain signals resulting from the stimulation cannot be identified in real time. Given the functionality of microchips for use in small systems, algorithms to detect biomarkers in real time can be embedded in a custom chip of a wearable system (148). Moreover, with embedded ML algorithms, multimodal EEG, fNIRS, and other related physiological recordings can be analyzed to identify signs of addiction more accurately. A support vector machine (SVM) algorithm has been implemented to stratify METH-user and healthy groups using FC network features of EEG signals (115). Concerning the relative frequency-specific power change ratio of EEG signals, SVM, logistic regression (LR), decision tree (DT), random forest (RF), multilayer perceptron, radial basis function networks, AdaBoost and gradian boost are implemented to compare the accuracy of classifying the METH and healthy groups (149). A convolutional neural network (CNN) model has been applied to EEG-fNIRS signals to classify METH addiction into light, moderate, and severe (93). Another CNN was used to classify the fNIRS signals of METH users and mixed users (150). The same research group compared the performances of linear discriminant analysis, SVM, and CNN of fNIRS signals to distinguish METH users and mixed users (87). When applying the EEG and GSR data, the accuracy of distinguishing the METH and the healthy group determined by random forest, logistic regression, and SVM have been compared (109).

Another limitation is that when ML models are designed for METH addiction applications, the performance of classification for different types of drugs or different periods of METH use is the focus, rather than the features used in stratification and the links between neurological systems. This limits our understanding of how these algorithms work, and thus, makes it hard to improve upon them. A more detailed interpretation of ML models would increase the confidence of clinical professionals in the classification results. Yet other limitation of existing EEG or fNIRS biomarkers is that they cannot be used to determine the stage or severity of addiction; therefore, they cannot be used to evaluate treatment effects during the therapy or as features to predict the possibility of relapse. Until now, the most used approach for stratifying the severity of addiction has been questionnaires, although the scores are based on subjective answers. Using body fluidic tests, the amount of drug in one’s system can be quantified. It has been shown that the longer a person uses METH, the stronger their ΔSCR reactions are to drug-related cues (70). As neuroimaging techniques are more complex than the above-mentioned methods, there have been limited studies on the correlations of the severity of addiction with the EEG or fNIRS biomarkers discussed in section “4.2. Biomarkers of EEG and fNIRS in METH addiction.” Conversely, the severity level (degree) of addiction during abstinence is also an issue that interests not only academics but also the judicial community (54). There is no solid evaluation system available to treatment centers to assess whether a patient has been rehabilitated when the scheduled therapy period is completed. With real-time signal-processing chips, various experimental protocols could be implemented to quantify the recorded biomarkers, providing information about the severity of addiction, amount of successful recovery, and correlations of these factors with cognitive functions.

#### 6.1.3. Portable/wearable TMS for customized treatments

The TMS protocol has not been extensively customized; previous studies have reported the effect of treatments in a fixed time frame owing to a pre-scheduled treatment protocol. To ensure a significant effect when comparing experimental and the controlled groups, which require analysis of performance over a fixed and limited time period, the protocols are often prescribed for a period from a few weeks to months (Table 5). Moreover, few studies have investigated the long-term effects of the treatment after the prescribed TMS treatment had been completed. These limitations of the published research are also due to the low accessibility of the commercially available bulky TMS devices and a lack of professional operators to conduct the treatment.

With the proposed wearable closed-loop system, treatment could be conducted at any time when it is needed. First, the parameters of the stimulation protocol (amplitude, frequency, intertrain interval, and duration) and numbers of treatment sessions can be customized depending on the real-time-monitored neural signals. Ideally, the potential customized protocol would increase the efficiency of the TMS devices and the work of clinical professionals. Second, the efficacy of TMS treatment could be further improved by the use of a wearable TMS system. A wearable TMS device could provide the required stimulations without constraints of time and location. However, the wearability of the TMS system depends strongly on miniaturization of the magnetic coil and the control module. Achieving sufficient stimulation voltage for neuromodulation applications is an important issue to be addressed in the development of a compact wearable TMS system (48).

### 6.2. Apply our closed-loop system to treat METH addiction

The treatment often has a predefined protocol in a rehabilitation center for drug abstinence. Patients receive a fixed amount of therapy in a fixed period. In addition, the effect of the treatment is difficult to quantify. This condition limits the flexibility of customizing the treatment protocol based on the latest conditions. One scenario of applying the closed-loop system in proposed.

#### 6.2.1. Baseline brain signal recording

The patients are recruited using EEG-fNIRS measurements to record the baseline brain signals. During the recording, the patients will be guided to watch the cues, such as drug-related pictures and videos, on a screen and rate the level of craving after seeing the cues. The differences of the various parameters of EEG and fNIRS signals from METH disorder users and healthy participants are analyzed. Moreover, various recorded signals when receiving neutral or drug-related cues are investigated. Brain signals’ features, found explicitly in patients receiving drug-related cues, are defined as biomarkers. During biomarker identification, no real addictive stimulus (drug) is required to arouse the thoughts of craving.

#### 6.2.2. Biomarkers identification to predict the desire of METH

During daily life, no matter in the rehabilitation centers or not, patients wear the EEG-fNIRS cap as frequently as possible. Twenty-four hours per day would be optimal. Theoretically, when the users receive anything related to their previous experiences of METH use, the brain signals alter, and specific signals containing biomarkers can be detected. The potential cues can be a wide variety, such as the environment similar to where the users used METH, the people who look identical to who the users had METH with, and the items with similar shapes or functions as the tools used for METH. The advantages of the real-time signal processing chips are that the biomarkers can be extracted and compared with baseline biomarkers at every moment. As soon as the results of biomarkers comparison can reveal the increasing of craving, an alarm will be sent to the TMS module to active a treatment. Moreover, with the low-power and high-performance real-time processing chips, multimodal biomarkers can be analyzed without delay.

#### 6.2.3. TMS protocol customization

If this is the first time the TMS is activated, the protocol applied by previous publications can be used. During the TMS planned period, the biomarker changes are identified when being exposed to the cues which bring more craving, seeing real drug, for example. If a second round of TMS can be conducted after the planned stimulation period and the variance of the biomarkers before and after the treatment is not apparent, this can be a sign to reduce the TMS treatment intensity or even stop the stimulation (Figure 1B). Thanks to the flexibility of the wearable TMS module, the optimal stimulation onset time when biomarkers are detected can be explored. Moreover, how long the TMS therapy last can be studied.

#### 6.2.4. Contributions to clinical practice and medicine

**FIGURE 5:**
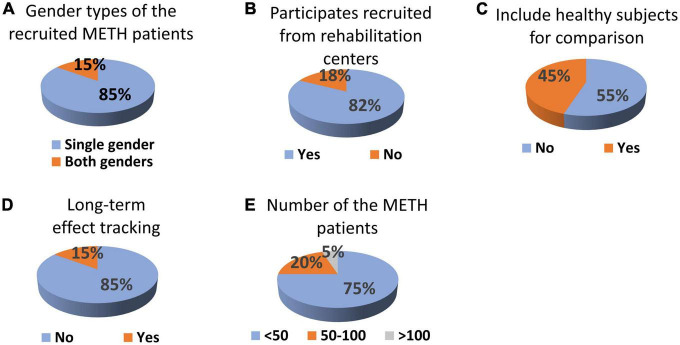
The summary of the limitations of literatures listed in Tables 1–4. **(A)** Gender types of the recruited subjects with METH addiction; **(B)** whether the participants are recruited from rehabilitation centers; **(C)** whether healthy subjects are included as comparison; **(D)** whether long-term effects are tracked; and **(E)** number of the subject with METH addiction in each study.

With this wearable closed-loop system, many research questions which were listed in the limitations of previous published work (Tables 1–4) can be more feasible to conduct. Figure 5 summarizes the main limitations of the literature reviewed in Tables 1–4 of this review article. In total, there are 40 pieces of literature included. As shown in Figure 5A, 34 papers have single genders, and 7 are females. Single-gender dominates because most experiments are conducted in rehabilitation centers where single-gender is allowed in most places. Figure 5B, only the studies that recruited participants from society have a chance to include both genders. Figure 5C, not all studies include healthy subjects as a comparison. When validating the effect of neuromodulation techniques, often, the stimulations are not conducted on healthy subjects due to ethical concerns. Excluding the literature aimed to explore the biomarkers of METH and healthy group, others implemented neuromodulations or therapies, such as exercise training. However, only 4 of the 27 studies performed the track monitoring to evaluate the long-term effect of the treatments (Figure 5D). For clinical studies, more subjects can lead to a more solid conclusion. Among the 40 pieces of literature in the tables of this review, 30 studies contain less than 50 METH patients, eight studies contain 50–100 METH patients, and two studies have over 100 METH patients. It is noted that even though some literature uses the same group of participants, they are still considered individual studies in Figure 5. These limitations and challenges to obtaining an optimal experiment are due to limited devices and manpower to conduct a large-scale investigation and later analyze the massive data.

The wearable closed-loop system we propose is highly accessible and user-friendly, and can be easily applied to a broader range of subjects. This means measurements on both genders and on both healthy and used disordered. Moreover, longitudinal recordings to track the long-term effect of the TMS treatment can be investigated. This system can provide a quantitative evaluation of the craving level in real-time. This is helpful for clinical doctors to adjust the therapy plan in the rehabilitation centers. Further quantitatively support the decision to leave the rehabilitation centers in advance or extend the stay. Furthermore, subjects not in the rehabilitation center, meaning those still using METH and those leaving the rehabilitation center, can be easily monitored. All scenarios, besides the rehabilitation centers, exit real METH. Therefore, the risk of relapse can be higher, which might result in more effective treatment of the system. With chips that can powerfully analyze the signals using ML algorithms in real-time, multimodal biomarkers can be analyzed efficiently.

We intend this review to provide a foundation for our current understanding of the potential for wearable closed-loop neuromodulation for treating methamphetamine addiction, which hopefully could be used for other addictive disorders. Here we detail wearable technologies and how those could be interfaced with neuroimaging techniques to understand how brain signals may relate to and influence biological signatures identified using the wearables. This information would be required to design closed-loop stimulation parameters that could be applied to patients. Our goal is that this review will provide a state of the field and a clear set of questions and next steps, including barriers that need to be overcome, to design such systems, which we believe hold great promise.

## 7. Conclusion

To boost the effectiveness of TMS treatment for METH addiction, we propose the concept of a wearable closed-loop neuromodulation with three main parts: a real-time brain signal monitoring system, an artificial intelligence signal processing system, and a customized neuromodulation system. In this review, we have summarized the research findings relevant to the essential modules required to achieve a wearable system that can be used to efficiently treat addiction, including biomarkers of EEG and fNIRS signals in patients with METH addiction, ML algorithms that can identify METH addiction, and applied TMS protocols to treat the addiction. Moreover, various cues that can be used to induce the desire for METH in validation experiments have been introduced. This novel approach currently focuses on METH but could be applied to other substance addictions in the future.

## Author contributions

Y-HC, JY, HW, and MS: conceptualization and writing—review and editing. Y-HC and JY: data curation. Y-HC, JY, and KB: writing—original draft preparation. Y-HC: visualization. MS: supervision, project administration, and funding acquisition. All authors contributed to the article and approved the submitted version.

## References

[1] Proebstl L Kamp F Koller G Soyka M. Cognitive deficits in methamphetamine users: how strong is the evidence? *Pharmacopsychiatry.* (2018) 51:243–50. 29334687 10.1055/s-0043-123471

[2] Paulus M Stewart J. Neurobiology, clinical presentation, and treatment of methamphetamine use disorder: a review. *JAMA Psychiatry.* (2020) 77:959–66. 10.1001/jamapsychiatry.2020.0246 32267484 PMC8098650

[3] Huang Q Chen X Huang S Shao T Liao Z Lin S Substance and internet use during the covid-19 pandemic in China. *Transl Psychiatry.* (2021) 11:491. 10.1038/s41398-021-01614-1 34556627 PMC8459580

[4] Karimi-Haghighi S Chavoshinezhad S Mozafari R Noorbakhsh F Borhani-Haghighi A Haghparast A. Neuroinflammatory response in reward-associated psychostimulants and opioids: a review. *Cell Mol Neurobiol.* (2022). 10.1007/s10571-022-01223-6 [Epub ahead of print]. 35461410 PMC11415174

[5] Altshuler R Lin H Li X. Neural mechanisms underlying incubation of methamphetamine craving: a mini-review. *Pharmacol Biochem Behav.* (2020) 199:173058. 10.1016/j.pbb.2020.173058 33250444 PMC7780755

[6] Tobolski J Sawyer D Song S Afari M. Cardiovascular disease associated with methamphetamine use: a review. *Heart Fail Rev.* (2022) 27:2059–65. 10.1007/s10741-022-10261-7 35844009

[7] Miller D Bu M Gopinath A Martinez L Khoshbouei H. Methamphetamine dysregulation of the central nervous system and peripheral immunity. *J Pharmacol Exp Ther.* (2021) 379:372. 10.1124/jpet.121.000767 34535563 PMC9351721

[8] May A Aupperle R Stewart J. Dark times: the role of negative reinforcement in methamphetamine addiction. *Front Psychiatry.* (2020) 11:114. 10.3389/fpsyt.2020.00114 32256392 PMC7090143

[9] De Berardis D Fornaro M Orsolini L Ventriglio A Vellante F Di Giannantonio M. Emotional dysregulation in adolescents: implications for the development of severe psychiatric disorders, substance abuse, and suicidal ideation and behaviors. *Brain Sci.* (2020) 10:591. 10.3390/brainsci10090591 PMC7565002 32858969

[10] Orsolini L Chiappini S Papanti D De Berardis D Corkery J Schifano F. The bridge between classical and “synthetic”/chemical psychoses: towards a clinical, psychopathological, and therapeutic perspective. *Front Psychiatry.* (2019) 10:851. 10.3389/fpsyt.2019.00851 31849723 PMC6896660

[11] Stellern J Xiao K Grennell E Sanches M Gowin J Sloan M. Emotion regulation in substance use disorders: a systematic review and meta-analysis. *Addiction.* (2022) 118:30–47. 10.1111/add.16001 35851975 PMC10087816

[12] Martinotti G Schiavone S Negri A Vannini C Trabace L De Berardis D Suicidal behavior and club drugs in young adults. *Brain Sci.* (2021) 11:490. 10.3390/brainsci11040490 PMC8069608 33921484

[13] Sanatkar S Heinsch M Tickner C Hunt S Teesson M Geddes J A systematic literature review and narrative synthesis of effective interventions for family and caregivers of people who use methamphetamine. *Subst Abuse.* (2022) 43:1190–6. 10.1080/08897077.2022.2074600 35617624

[14] Zhang B Yan X Li Y Zhu H Lu Z Jia Z. Trends in methamphetamine use in the Mainland of China, 2006–2015. *Front Public Health.* (2022) 10:852837. 10.3389/fpubh.2022.852837 35570894 PMC9096246

[15] United Nations. *Synthetic Drugs in East and Southeast Asia: Latest Developments and Challenges 2022.* New York, NY: United Nations (2022).

[16] Hamel C Corace K Hersi M Rice D Willows M Macpherson P Psychosocial and pharmacologic interventions for methamphetamine addiction: protocol for a scoping review of the literature. *Syst Rev.* (2020) 9:245. 10.1186/s13643-020-01499-z 33099314 PMC7585172

[17] Moszczynska A. Current and emerging treatments for methamphetamine use disorder. *Curr Neuropharmacol.* (2021) 19:2077–91. 10.2174/1570159x19666210803091637 34344291 PMC9185770

[18] Tran M Luong Q Le Minh G Dunne M Baker P. Psychosocial interventions for amphetamine type stimulant use disorder: an overview of systematic reviews. *Front Psychiatry.* (2021) 12:512076. 10.3389/fpsyt.2021.512076 34220557 PMC8245759

[19] Newman A Ku T Jordan C Bonifazi A Xi Z. New drugs, old targets: tweaking the dopamine system to treat psychostimulant use disorders. *Annu Rev Pharmacol Toxicol.* (2021) 61:609–28. 10.1146/annurev-pharmtox-030220-124205 33411583 PMC9341034

[20] Härtel-Petri R Krampe-Scheidler A Braunwarth W Havemann-Reinecke U Jeschke P Looser W Evidence-based guidelines for the pharmacologic management of methamphetamine dependence, relapse prevention, chronic methamphetamine-related, and comorbid psychiatric disorders in post-acute settings. *Pharmacopsychiatry.* (2017) 50:96–104. Epub 26.04.2017. 28445899 10.1055/s-0043-105500

[21] Khorablou Z Shahdost-fard F Razmi H Yola M Karimi-Maleh H. Recent advances in developing optical and electrochemical sensors for analysis of methamphetamine: a review. *Chemosphere.* (2021) 278:130393. 10.1016/j.chemosphere.2021.130393 33823350

[22] Martini M Batista T Henn I Souza P Vieira A Azevedo-Alanis L. Whether drug detection in urine and oral fluid is similar? a systematic review. *Crit Rev Toxicol.* (2020) 50:348–58. 10.1080/10408444.2020.1751062 32343161

[23] De Rycke E Stove C Dubruel P De Saeger S Beloglazova N. Recent developments in electrochemical detection of illicit drugs in diverse matrices. *Biosens Bioelectron.* (2020) 169:112579. 10.1016/j.bios.2020.112579 32947080

[24] Kuwayama K Miyaguchi H Kanamori T Tsujikawa K Yamamuro T Segawa H Micro-segmental hair analysis: detailed procedures and applications in forensic toxicology. *Forensic Toxicol.* (2022) 40:215–33. 10.1007/s11419-022-00619-9 36454411 PMC9715473

[25] Hendianti G Uthis P. Factors related to methamphetamine relapse risk among clients in the substance rehabilitation center of national narcotics board in West Java, Indonesia. *J Health Res.* (2018) 32:279–87. 10.1108/JHR-05-2018-035

[26] Xu S Zhang K Luo T. Development of the risk of relapse assessment scale for methamphetamine abusers in China. *Drug Alcohol Depend.* (2021) 227:108992. 10.1016/j.drugalcdep.2021.108992 34482042

[27] Mahmud M Fang H Carreiro S Wang H Boyer E. Wearables Technology for drug abuse detection: a survey of recent advancement. *Smart Health.* (2019) 13:100062. 10.1016/j.smhl.2018.09.002

[28] Carreiro S Fang H Zhang J Wittbold K Weng S Mullins R Imstrong: deployment of a biosensor system to detect cocaine use. *J Med Syst.* (2015) 39:186. 10.1007/s10916-015-0337-9 26490144 PMC4888957

[29] Tisdall L MacNiven K Padula C Leong J Knutson B. Brain tract structure predicts relapse to stimulant drug use. *Proc Natl Acad Sci.* (2022) 119:e2116703119. 10.1073/pnas.2116703119 35727973 PMC9245633

[30] Parvaz M Rabin R Adams F Goldstein R. Structural and functional brain recovery in individuals with substance use disorders during abstinence: a review of longitudinal neuroimaging studies. *Drug Alcohol Depend.* (2022) 232:109319. 10.1016/j.drugalcdep.2022.109319 35077955 PMC8885813

[31] Chen C Hsu F Li C Huang M. Structural, functional, and neurochemical neuroimaging of methamphetamine-associated psychosis: a systematic review. *Psychiatry Res.* (2019) 292:23–31. 10.1016/j.pscychresns.2019.06.002 31476712

[32] Sabrini S Wang G Lin J Ian J Curley L. Methamphetamine use and cognitive function: a systematic review of neuroimaging research. *Drug Alcohol Depend.* (2019) 194:75–87. 10.1016/j.drugalcdep.2018.08.041 30414539

[33] Stewart J May A Paulus M. Bouncing back: brain rehabilitation amid opioid and stimulant epidemics. *NeuroImage.* (2019) 24:102068. 10.1016/j.nicl.2019.102068 31795056 PMC6978215

[34] Qi L Tian Z Yue Y Guan S Tang L Dong G. Effects of acute exercise on craving and cortical hemodynamics under drug-cue exposure in ma-dependent individuals. *Neurosci Lett.* (2022) 781:136672. 10.1016/j.neulet.2022.136672 35504405

[35] Chen Y Lu Y Zhou C Wang X. The effects of aerobic exercise on working memory in methamphetamine-dependent patients: evidence from combined Fnirs and Erp. *Psychol Sport Exerc.* (2020) 49:101685. 10.1016/j.psychsport.2020.101685

[36] Moeller S Paulus M. Toward biomarkers of the addicted human brain: using neuroimaging to predict relapse and sustained abstinence in substance use disorder. *Prog Neuropsychopharmacol Biol Psychiatry.* (2018) 80:143–54. 10.1016/j.pnpbp.2017.03.003 28322982 PMC5603350

[37] Song S Zilverstand A Gui W Li HJ Zhou X. Effects of single-session versus multi-session non-invasive brain stimulation on craving and consumption in individuals with drug addiction, eating disorders or obesity: a meta-analysis. *Brain Stimul.* (2019) 12:606–18. 10.1016/j.brs.2018.12.975 30612944

[38] Mahoney J Hanlon C Marshalek P Rezai A Krinke L. Transcranial magnetic stimulation, deep brain stimulation, and other forms of neuromodulation for substance use disorders: review of modalities and implications for treatment. *J Neurol Sci.* (2020) 418:117149. 10.1016/j.jns.2020.117149 33002757 PMC7702181

[39] Song S Zilverstand A Gui W Pan X Zhou X. Reducing craving and consumption in individuals with drug addiction, obesity or overeating through neuromodulation intervention: a systematic review and meta-analysis of its follow-up effects. *Addiction.* (2022) 117:1242–55. 10.1111/add.15686 34514666

[40] Chang R Peng J Chen Y Liao H Zhao S Zou J Deep brain stimulation in drug addiction treatment: research progress and perspective. *Front Psychiatry.* (2022) 13:858638. 10.3389/fpsyt.2022.858638 35463506 PMC9022905

[41] Moretti J Poh E Rodger J. Rtms-induced changes in glutamatergic and dopaminergic systems: relevance to cocaine and methamphetamine use disorders. *Front Neurosci.* (2020) 14:137. 10.3389/fnins.2020.00137 32210744 PMC7068681

[42] Jiang X Tian Y Zhang Z Zhou C Yuan J. The counterproductive effect of right anodal/left cathodal transcranial direct current stimulation over the dorsolateral prefrontal cortex on impulsivity in methamphetamine addicts. *Front Psychiatry.* (2022) 13:915440. 10.3389/fpsyt.2022.915440 35815052 PMC9257135

[43] Figeys M Zeeman M Kim E. Effects of transcranial direct current stimulation (Tdcs) on cognitive performance and cerebral oxygen hemodynamics: a systematic review. *Front Hum Neurosci.* (2021) 15:623315. 10.3389/fnhum.2021.623315 33897392 PMC8058208

[44] Zhong G Yang Z Jiang T. Precise modulation strategies for transcranial magnetic stimulation: advances and future directions. *Neurosci Bull.* (2021) 37:1718–34. 10.1007/s12264-021-00781-x 34609737 PMC8643289

[45] Salling M Martinez D. Brain stimulation in addiction. *Neuropsychopharmacology.* (2016) 41:2798–809. 10.1038/npp.2016.80 27240657 PMC5061891

[46] Lloyd J Hill B Murphy M Al-Kaisy A Andreou A Lambru G. Single-Pulse transcranial magnetic stimulation for the preventive treatment of difficult-to-treat migraine: a 12-month prospective analysis. *J Headache Pain.* (2022) 23:63. 10.1186/s10194-022-01428-6 35668368 PMC9169440

[47] Lee S Jang K Yoon S Chae J. The efficacy of miniaturized repetitive transcranial magnetic stimulation in patients with depression. *Clin Psychopharmacol Neurosci.* (2019) 17:409–14. 10.9758/cpn.2019.17.3.409 31352707 PMC6705096

[48] Li N Yang J Sawan M editors. Compact closed-loop Eeg/Fnirs recording and tms neuromodulation system. *Proceedings of the 2022 20th IEEE Interregional NEWCAS Conference (NEWCAS).* Québec, CA: (2022).

[49] National Institute on Drug Abuse. *Screening and Assessment Tools Chart.* Bethesda, MD: National Institute on Drug Abuse (2021).

[50] Skinner H. The drug abuse screening test. *Addict Behav.* (1982) 7:363–71. 10.1016/0306-4603(82)90005-3 7183189

[51] National Institute on Drug Abuse. *Diagnosis and treatment of drug abuse in family practice – american family physician monograph.* Bethesda, MD: National Institute on Drug Abuse (2003).

[52] Franken I Hendriks V van den Brink W. Initial validation of two opiate craving questionnaires: the obsessive compulsive drug use scale and the desires for drug questionnaire. *Addict Behav.* (2002) 27:675–85. 10.1016/S0306-4603(01)00201-5 12201376

[53] McLellan A Luborsky L Woody G O’Brien C. An improved diagnostic evaluation instrument for substance abuse patients. The addiction severity index. *J Nerv Ment Dis.* (1980) 168:26–33. 10.1097/00005053-198001000-00006 7351540

[54] Zhao D Zhang M Tian W Cao X Yin L Liu Y Neurophysiological correlate of incubation of craving in individuals with methamphetamine use disorder. *Mol Psychiatry.* (2021) 26:6198–208. 10.1038/s41380-021-01252-5 34385601

[55] Hiller M Broome K Knight K Simpson D. Measuring self-efficacy among drug-involved probationers. *Psychol Rep.* (2000) 86:529–38. 10.2466/pr0.2000.86.2.529 10840908

[56] Handelsman L Cochrane K Aronson M Ness R Rubinstein K Kanof P. Two new rating scales for opiate withdrawal. *Am J Drug Alcohol Abuse.* (1987) 13:293–308. 10.3109/00952998709001515 3687892

[57] Johnson B Ait-Daoud N Elkashef A Smith E Kahn R Vocci F A preliminary randomized, double-blind, placebo-controlled study of the safety and efficacy of ondansetron in the treatment of methamphetamine dependence. *Int J Neuropsychopharmacol.* (2008) 11:1–14. 10.1017/S1461145707007778 17470315

[58] Ogai Y Haraguchi A Kondo A Ishibashi Y Umeno M Kikumoto H Development and validation of the stimulant relapse risk scale for drug abusers in Japan. *Drug Alcohol Depend.* (2007) 88:174–81. 10.1016/j.drugalcdep.2006.10.005 17118576

[59] Adinoff B Talmadge C Williams M Schreffler E Jackley P Krebaum S. Time to relapse questionnaire (Trq): a measure of sudden relapse in substance dependence. *Am J Drug Alcohol Abuse.* (2010) 36:140–9. 10.3109/00952991003736363 20465371 PMC3600377

[60] Khazaee-Pool M Moridi M Ponnet K Turner N Pashaei T. Psychometric properties of the persian version of the time to relapse questionnaire (Trq) in substance use disorder. *Am J Drug Alcohol Abuse.* (2016) 42:682–8. 27286097 10.3109/00952990.2016.1172593

[61] Liang Q Yuan T Cao X He H Yang J Yuan J. Assessing the severity of methamphetamine use disorder beyond the subjective craving report: the role of an attention bias test. *Gen Psychiatry.* (2019) 32:e100019. 10.1136/gpsych-2018-100019 31179431 PMC6551440

[62] Palmeri A Pichini S Pacifici R Zuccaro P Lopez A. Drugs in nails: physiology, pharmacokinetics and forensic toxicology. *Clin Pharmacokinet.* (2000) 38:95–110. 10.2165/00003088-200038020-00001 10709775

[63] Palamar J Le A Guarino H Mateu-Gelabert PA. Comparison of the utility of urine- and hair testing in detecting self-reported drug use among young adult opioid users. *Drug Alcohol Depend.* (2019) 200:161–7. 10.1016/j.drugalcdep.2019.04.008 31146203 PMC6588496

[64] Trefz P Kamysek S Fuchs P Sukul P Schubert J Miekisch W. Drug detection in breath: non-invasive assessment of illicit or pharmaceutical drugs. *J Breath Res.* (2017) 11:024001. 10.1088/1752-7163/aa61bf 28220762

[65] Goldfine C Lai J Lucey E Newcomb M Carreiro S. Wearable and wireless mhealth technologies for substance use disorder. *Curr Addict Rep.* (2020) 7:291–300. 10.1007/s40429-020-00318-8 33738178 PMC7963000

[66] Teymourian H Parrilla M Sempionatto J Montiel N Barfidokht A Van Echelpoel R Wearable electrochemical sensors for the monitoring and screening of drugs. *ACS Sens.* (2020) 5:2679–700. 10.1021/acssensors.0c01318 32822166

[67] Gullapalli B Natarajan A Angarita G Malison R Ganesan D Rahman T. On-body sensing of cocaine craving, euphoria and drug-seeking behavior using cardiac and respiratory signals. *Proc ACM Interact Mob Wearable Ubiquitous Technol.* (2019) 3:31. 10.1145/3328917

[68] Carreiro S Smelson D Ranney M Horvath K Picard R Boudreaux Real-time mobile detection of drug use with wearable biosensors: a pilot study. *J Med Toxicol.* (2015) 11:73–9. 10.1007/s13181-014-0439-7 25330747 PMC4371024

[69] Wang Y Shen Z Wu X. Detection of patients with methamphetamine dependence with cue-elicited heart rate variability in a virtual social environment. *Psychiatry Res.* (2018) 270:382–8. 10.1016/j.psychres.2018.10.009 30300868

[70] Guerin A Drummond K Bonomo Y Lawrence A Rossell S Kim J. Assessing methamphetamine-related cue reactivity in people with methamphetamine use disorder relative to controls. *Addict Behav.* (2021) 123:107075. 10.1016/j.addbeh.2021.107075 34385074

[71] Liu X Wang S. Effect of aerobic exercise on executive function in individuals with methamphetamine use disorder: modulation by the autonomic nervous system. *Psychiatry Res.* (2021) 306:114241. 10.1016/j.psychres.2021.114241 34688059

[72] Dhingra D Kaur S Ram J. Illicit drugs: effects on eye. *Indian J Med Res.* (2019) 150:228–38. 10.4103/ijmr.IJMR_1210_17 31719293 PMC6886135

[73] Tsai M Chung C Chen C Chen J Yeh S Lin C An intelligent virtual-reality system with multi-model sensing for cue-elicited craving in patients with methamphetamine use disorder. *IEEE Trans Biomed Eng.* (2021) 68:2270–80. 10.1109/tbme.2021.3058805 33571085

[74] Carreiro S Chintha K Shrestha S Chapman B Smelson D Indic P. Wearable sensor-based detection of stress and craving in patients during treatment for substance use disorder: a mixed methods pilot study. *Drug Alcohol Depend.* (2020) 209:107929. 10.1016/j.drugalcdep.2020.107929 32193048 PMC7197459

[75] Epstein D Tyburski M Kowalczyk W Burgess-Hull A Phillips K Curtis B Prediction of stress and drug craving ninety minutes in the future with passively collected Gps data. *NPJ Digit Med.* (2020) 3:26. 10.1038/s41746-020-0234-6 32195362 PMC7055250

[76] Parvaz M Alia-Klein N Woicik P Volkow N Goldstein R. Neuroimaging for drug addiction and related behaviors. *Rev Neurosci.* (2011) 22:609–24. 10.1515/RNS.2011.055 22117165 PMC3462350

[77] Hayes A Herlinger K Paterson L Lingford-Hughes A. The neurobiology of substance use and addiction: evidence from neuroimaging and relevance to treatment. *BJPsych Adv.* (2020) 26:367–78. 10.1192/bja.2020.68

[78] Zilverstand A Huang A Alia-Klein N Goldstein R. Neuroimaging impaired response inhibition and salience attribution in human drug addiction: a systematic review. *Neuron.* (2018) 98:886–903. 10.1016/j.neuron.2018.03.048 29879391 PMC5995133

[79] Garrison K Potenza M. Neuroimaging and biomarkers in addiction treatment. *Curr Psychiatry Rep.* (2014) 16:513. 10.1007/s11920-014-0513-5 25308385 PMC4427893

[80] Stewart J May A Aupperle R Bodurka J. Forging neuroimaging targets for recovery in opioid use disorder. *Front Psychiatry.* (2019) 10:117. 10.3389/fpsyt.2019.00117 30899231 PMC6417368

[81] London ED Kohno M Morales A Ballard M. Chronic methamphetamine abuse and corticostriatal deficits revealed by neuroimaging. *Brain Res.* (2015) 1628(Pt A):174–85. 10.1016/j.brainres.2014.10.044 25451127 PMC4418947

[82] Rathitharan G Truong J Tong J McCluskey T Meyer J Mizrahi R Microglia imaging in methamphetamine use disorder: a positron emission tomography study with the 18 Kda translocator protein radioligand [F-18]Feppa. *Addict Biol.* (2021) 26:e12876. 10.1111/adb.12876 32017280 PMC7398821

[83] Lai X Huang Q Xin J Yu H Wen J Huang S Identifying methamphetamine abstainers with convolutional neural networks and short-time fourier transform. *Front Psychol.* (2021) 12:684001. 10.3389/fpsyg.2021.684001 34456796 PMC8385271

[84] Sanjari Moghaddam H Mobarak Abadi M Dolatshahi M Bayani Ershadi S Abbasi-Feijani F Rezaei S Effects of prenatal methamphetamine exposure on the developing human brain: a systematic review of neuroimaging studies. *ACS Chem Neurosci.* (2021) 12:2729–48. 10.1021/acschemneuro.1c00213 34297546 PMC8763371

[85] Lu Y Qi X Zhao Q Chen Y Liu Y Li X Effects of exercise programs on neuroelectric dynamics in drug addiction. *Cogn Neurodyn.* (2021) 15:27–42. 10.1007/s11571-020-09647-w 33786077 PMC7947158

[86] Li R Yang D Fang F Hong K Reiss A Zhang Y. Concurrent Fnirs and Eeg for brain function investigation: a systematic, methodology-focused review. *Sensors.* (2022) 22:5865. 10.3390/s22155865 35957421 PMC9371171

[87] Gu X Yang B Gao S Yan L Xu D Wang W. Prefrontal Fnirs-based clinical data analysis of brain functions in individuals abusing different types of drugs. *J Biomed Semantics.* (2021) 12:21. 10.1186/s13326-021-00256-y 34823598 PMC8620253

[88] Bu L Qi L Yan W Yan Q Tang Z Li F Acute kick-boxing exercise alters effective connectivity in the brain of females with methamphetamine dependencies. *Neurosci Lett.* (2020) 720:134780. 10.1016/j.neulet.2020.134780 31978497

[89] Bu L Wu Y Yan Q Tang L Liu X Diao C Effects of physical training on brain functional connectivity of methamphetamine dependencies as assessed using functional near-infrared spectroscopy. *Neurosci Lett.* (2020) 715:134605. 10.1016/j.neulet.2019.134605 31698028

[90] Zhou Y Finlayson G Liu X Zhou Q Liu T Zhou C. Effects of acute dance and aerobic exercise on drug craving and food reward in women with methamphetamine dependence. *Med Sci Sports Exerc.* (2021) 53:2245–53. 10.1249/mss.0000000000002723 34115731

[91] Yang B Gu X Gao S Xu D. Classification accuracy and functional difference prediction in different brain regions of drug abuser prefrontal lobe basing on machine-learning. *Math Biosci Eng.* (2021) 18:5692–706. 10.3934/mbe.2021288 34517508

[92] Yang B Gu X Xu D Fan C. A study on eeg-nirs testing of drug users’ brain function under visual induction. *Proceedings of the 2020 9th International Conference on Computing and Pattern Recognition.* Xiamen: Association for Computing Machinery (2020). p. 85–9.

[93] Gu X Yang B Gao S Yan L Xu D Wang W. Application of Bi-modal signal in the classification and recognition of drug addiction degree based on machine learning. *Math Biosci Eng.* (2021) 18:6926–40. 10.3934/mbe.2021344 34517564

[94] Ieong H Gao F Yuan Z. Machine learning: assessing neurovascular signals in the prefrontal cortex with non-invasive bimodal electro-optical neuroimaging in opiate addiction. *Sci Rep.* (2019) 9:18262. 10.1038/s41598-019-54316-6 31797878 PMC6892956

[95] Habelt B Arvaneh M Bernhardt N Minev I. Biomarkers and neuromodulation techniques in substance use disorders. *Bioelectron Med.* (2020) 6:4. 10.1186/s42234-020-0040-0 32232112 PMC7098236

[96] Seow L Ong W Hombali A AshaRani P Subramaniam M. A scoping review on cue reactivity in methamphetamine use disorder. *Int J Environ Res Public Health.* (2020) 17:6504. 10.3390/ijerph17186504 32906716 PMC7558044

[97] Culbertson C Nicolas S Zaharovits I London E De La Garza R Brody A Methamphetamine craving induced in an online virtual reality environment. *Pharmacol Biochem Behav.* (2010) 96:454–60. 10.1016/j.pbb.2010.07.005 20643158 PMC2951480

[98] Mazza M Kammler-Sücker K Leménager T Kiefer F Lenz B. Virtual reality: a powerful technology to provide novel insight into treatment mechanisms of addiction. *Transl Psychiatry.* (2021) 11:617. 10.1038/s41398-021-01739-3 34873146 PMC8648903

[99] Versace F Engelmann J Deweese M Robinson J Green C Lam C Beyond cue reactivity: non-drug-related motivationally relevant stimuli are necessary to understand reactivity to drug-related cues. *Nicotine Tob Res.* (2017) 19:663–9. 10.1093/ntr/ntx002 28486715 PMC5423099

[100] Ekhtiari H Kuplicki R Pruthi A Paulus M. Methamphetamine and opioid cue database (Mocd): development and validation. *Drug Alcohol Depend.* (2020) 209:107941. 10.1016/j.drugalcdep.2020.107941 32146357

[101] Huang J Zheng Y Gao D Hu M Yuan T. Effects of exercise on depression, anxiety, cognitive control, craving, physical fitness and quality of life in methamphetamine-dependent patients. *Front Psychiatry.* (2020) 10:999. 10.3389/fpsyt.2019.00999 32047445 PMC6997340

[102] Chen T Su H Zhong N Tan H Li X Meng Y Disrupted brain network dynamics and cognitive functions in methamphetamine use disorder: insights from Eeg microstates. *BMC Psychiatry.* (2020) 20:334. 10.1186/s12888-020-02743-5 32580716 PMC7315471

[103] Liu Y Chen Y Fraga-González G Szpak V Laverman J Wiers R Resting-State Eeg, substance use and abstinence after chronic use: a systematic review. *Clin EEG Neurosci.* (2022) 53:344–66. 10.1177/15500594221076347 35142589

[104] Newton T Cook I Kalechstein A Duran S Monroy F Ling W Quantitative Eeg abnormalities in recently abstinent methamphetamine dependent individuals. *Clin Neurophysiol.* (2003) 114:410–5. 10.1016/S1388-2457(02)00409-1 12705421

[105] Newton T Kalechstein A Hardy D Cook I Nestor L Ling W Association between quantitative Eeg and neurocognition in methamphetamine-dependent volunteers. *Clin Neurophysiol.* (2004) 115:194–8. 10.1016/S1388-2457(03)00314-6 14706488

[106] Yun K Park H Kwon D Cho S Jeong J editors. Decreased complexity of the Eeg in patients with methamphetamine dependence. In: Magjarevic R Nagel JH editors. *World Congress on Medical Physics and Biomedical Engineering 2006.* Berlin: Springer Berlin Heidelberg (2007).

[107] Kalechstein A De La Garza R Newton T Green M Cook I Leuchter A. Quantitative Eeg abnormalities are associated with memory impairment in recently abstinent methamphetamine-dependent individuals. *J Neuropsychiatry Clin Neurosci.* (2009) 21:254–8. 10.1176/jnp.2009.21.3.254 19776303 PMC3350803

[108] Howells F Temmingh H Hsieh J van Dijen A Baldwin D Stein D. Electroencephalographic Delta/Alpha frequency activity differentiates psychotic disorders: a study of schizophrenia, bipolar disorder and methamphetamine-induced psychotic disorder. *Transl Psychiatry.* (2018) 8:75. 10.1038/s41398-018-0105-y 29643331 PMC5895848

[109] Ding X Li Y Li D Li L Liu X. Using machine-learning approach to distinguish patients with methamphetamine dependence from healthy subjects in a virtual reality environment. *Brain Behav.* (2020) 10:e01814. 10.1002/brb3.1814 32862513 PMC7667292

[110] Minnerly C Shokry I To W Callanan J Tao R. Characteristic changes in Eeg spectral powers of patients with opioid-use disorder as compared with those with methamphetamine- and alcohol-use disorders. *PLoS One.* (2021) 16:e0248794. 10.1371/journal.pone.0248794 34506492 PMC8432824

[111] Shahmohammadi F Golesorkhi M Riahi Kashani M Sangi M Yoonessi A Yoonessi A. Neural correlates of craving in methamphetamine abuse. *Basic Clin Neurosci.* (2016) 7:221–30. 10.15412/J.BCN.03070307 27563415 PMC4981834

[112] Khajehpour H Parvaz M Kouti M Hosseini Rafsanjani T Ekhtiari H Bakht S Effects of transcranial direct current stimulation on attentional bias to methamphetamine cues and its association with Eeg-derived functional brain network topology. *Int J Neuropsychopharmacol.* (2022) 25:631–44. 10.1093/ijnp/pyac018 35380672 PMC9380716

[113] Ahmadlou M Ahmadi K Rezazade M Azad-Marzabadi E. Global organization of functional brain connectivity in methamphetamine abusers. *Clin Neurophysiol.* (2013) 124:1122–31. 10.1016/j.clinph.2012.12.003 23332777

[114] Khajehpour H Makkiabadi B Ekhtiari H Bakht S Noroozi A Mohagheghian F. Disrupted resting-state brain functional network in methamphetamine abusers: a brain source space study by Eeg. *PLoS One.* (2019) 14:e0226249. 10.1371/journal.pone.0226249 31825996 PMC6906079

[115] Khajehpour H Mohagheghian F Ekhtiari H Makkiabadi B Jafari A Eqlimi E Computer-aided classifying and characterizing of methamphetamine use disorder using resting-state Eeg. *Cogn Neurodyn.* (2019) 13:519–30. 10.1007/s11571-019-09550-z 31741689 PMC6825232

[116] Shafiee-Kandjani A Jahan A Moghadam-Salimi M Fakhari A Nazari M Sadeghpour S. Resting-state electroencephalographic coherence in recently abstinent methamphetamine users. *Int J High Risk Behav Addict.* (2020) 9:e103606. 10.5812/ijhrba.103606

[117] Qi X Wang Y Lu Y Zhao Q Chen Y Zhou C Enhanced brain network flexibility by physical exercise in female methamphetamine users. *Cogn Neurodyn.* (2022). 10.1007/s11571-022-09848-5 [Epub ahead of print]. PMC11655724 39712117

[118] Lin Q Li D Hu C Shen Z Wang Y. Altered Eeg microstates dynamics during cue-induced methamphetamine craving in virtual reality environments. *Front Psychiatry.* (2022) 13:891719. 10.3389/fpsyt.2022.891719 35599773 PMC9114476

[119] Wang H Chen Y Li X Wang J Zhou Y Zhou C. Moderate-Intensity aerobic exercise restores appetite and prefrontal brain activity to images of food among persons dependent on methamphetamine: a functional near-infrared spectroscopy study. *Front Hum Neurosci.* (2019) 13:400. 10.3389/fnhum.2019.00400 31798434 PMC6863778

[120] Tao Q Zhang C Li X. Dancing improves emotional regulation in women with methamphetamine use disorder but use of a cycle ergometer does not. *Front Neurosci.* (2021) 15:629061. 10.3389/fnins.2021.629061 34276278 PMC8282196

[121] Gao S Zhou C Chen Y. Effects of acute moderate- and high-intensity aerobic exercise on oxygenation in prefrontal cortex of male methamphetamine-dependent patients. *Front Psychol.* (2022) 13:801531. 10.3389/fpsyg.2022.801531 35153956 PMC8826552

[122] Qi L Yin Y Bu L Tang Z Tang L Dong G. Acute Vr competitive cycling exercise enhanced cortical activations and brain functional network efficiency in ma-dependent individuals. *Neurosci Lett.* (2021) 757:135969. 10.1016/j.neulet.2021.135969 34023411

[123] West R Hao W Lam T Lau J Li J Li J Addiction in China: towards a research agenda for the next 5 years. *Addiction.* (2019) 114:1911–4. 10.1111/add.14650 31081567

[124] Yuen J Kouzani A Berk M Tye S Rusheen A Blaha C Deep brain stimulation for addictive disorders—where are we now? *Neurotherapeutics.* (2022) 19:1193–215. 10.1007/s13311-022-01229-4 35411483 PMC9587163

[125] Chen T Su H Li R Jiang H Li X Wu Q The exploration of optimized protocol for repetitive transcranial magnetic stimulation in the treatment of methamphetamine use disorder: a randomized sham-controlled study. *EBioMedicine.* (2020) 60:103027. 10.1016/j.ebiom.2020.103027 32980696 PMC7522737

[126] Bikson M Datta A Rahman A Scaturro J. Electrode montages for Tdcs and weak transcranial electrical stimulation: role of “return” electrode’s position and size. *Clin Neurophysiol.* (2010) 121:1976–8. 10.1016/j.clinph.2010.05.020 21035740 PMC2983105

[127] Iyer M Mattu U Grafman J Lomarev M Sato S Wassermann E. Safety and cognitive effect of frontal dc brain polarization in healthy individuals. *Neurology.* (2005) 64:872. 10.1212/01.WNL.0000152986.07469.E9 15753425

[128] Shahbabaie A Golesorkhi M Zamanian B Ebrahimpoor M Keshvari F Nejati V State dependent effect of transcranial direct current stimulation (Tdcs) on methamphetamine craving. *Int J Neuropsychopharmacol.* (2014) 17:1591–8. 10.1017/S1461145714000686 24825251

[129] Shariatirad S Vaziri A Hassani-Abharian P Sharifi Fardshad M Molavi N Fitzgerald P. Cumulative and booster effects of tdcs sessions on drug cravings, lapse, and cognitive impairment in methamphetamine use disorder: a case study report. *Am J Addict.* (2016) 25:264–6. 10.1111/ajad.12373 27219624

[130] Shahbabaie A Ebrahimpoor M Hariri A Nitsche M Hatami J Fatemizadeh E Transcranial Dc stimulation modifies functional connectivity of large-scale brain networks in abstinent methamphetamine users. *Brain Behav.* (2018) 8:e00922. 10.1002/brb3.922 29541538 PMC5840443

[131] Anaraki M Dolatshahi B Nosratabadi M Yalghouzaghaji M Mashhadi S. A clinical trial: repeated transcranial direct current stimulation (Tdcs) on methamphetamine craving: a randomized, sham-controlled study. *Iran Rehabil J.* (2019) 17:385–94. 10.32598/irj.17.4.385

[132] Xu X Ding X Chen L Chen T Su H Li X The transcranial direct current stimulation over prefrontal cortex combined with the cognitive training reduced the cue-induced craving in female individuals with methamphetamine use disorder: a randomized controlled trial. *J Psychiatr Res.* (2021) 134:102–10. 10.1016/j.jpsychires.2020.12.056 33383492

[133] Tang Z Zhu Z Xu J. Psychological effects of repetitive transcranial magnetic stimulation on individuals with methamphetamine use disorder: a systematic review and meta-analysis. *Biol Res Nurs.* (2022) 25:117–28. 10.1177/10998004221122522 35999040

[134] Chang C Liou M Liu C Lu W Chen S. Efficacy of repetitive transcranial magnetic stimulation in patients with methamphetamine use disorder: a systematic review and meta-analysis of double-blind randomized controlled trials. *Front Psychiatry.* (2022) 13:904252. 10.3389/fpsyt.2022.904252 35711590 PMC9197111

[135] Zhao D Li Y Liu T Voon V Yuan T. Twice-daily theta burst stimulation of the dorsolateral prefrontal cortex reduces methamphetamine craving: a pilot study. *Front Neurosci.* (2020) 14:208. 10.3389/fnins.2020.00208 32273837 PMC7113524

[136] Zhang Y Ku Y Sun J Daskalakis Z Yuan T. Intermittent theta burst stimulation to the left dorsolateral prefrontal cortex improves working memory of subjects with methamphetamine use disorder. *Psychol Med.* (2021) 2021:1–10. 10.1017/S003329172100430X 37310309

[137] Wen Y Hao X Chen X Qiao S Li Q Winkler M Theta-burst stimulation combined with virtual-reality reconsolidation intervention for methamphetamine use disorder: study protocol for a randomized-controlled trial. *Front Psychiatry.* (2022) 13:903242. 10.3389/fpsyt.2022.903242 35865301 PMC9294395

[138] Gay A Cabe J De Chazeron I Lambert C Defour M Bhoowabul V Repetitive transcranial magnetic stimulation (Rtms) as a promising treatment for craving in stimulant drugs and behavioral addiction: a meta-analysis. *J Clin Med.* (2022) 11:624. 10.3390/jcm11030624 PMC8836499 35160085

[139] Zhang J Fong K Ouyang R Siu A Kranz G. Effects of repetitive transcranial magnetic stimulation (Rtms) on craving and substance consumption in patients with substance dependence: a systematic review and meta-analysis. *Addiction.* (2019) 114:2137–49. 10.1111/add.14753 31328353

[140] Ma T Sun Y Ku Y. Effects of non-invasive brain stimulation on stimulant craving in users of cocaine, amphetamine, or methamphetamine: a systematic review and meta-analysis. *Front Neurosci.* (2019) 13:1095. 10.3389/fnins.2019.01095 31680830 PMC6813242

[141] Li X Malcolm R Huebner K Hanlon C Taylor J Brady K Low frequency repetitive transcranial magnetic stimulation of the left dorsolateral prefrontal cortex transiently increases cue-induced craving for methamphetamine: a preliminary study. *Drug Alcohol Depend.* (2013) 133:641–6. 10.1016/j.drugalcdep.2013.08.012 24028801 PMC4196687

[142] Liang Y Wang L Yuan T. Targeting withdrawal symptoms in men addicted to methamphetamine with transcranial magnetic stimulation: a randomized clinical trial. *JAMA Psychiatry.* (2018) 75:1199–201. 10.1001/jamapsychiatry.2018.2383 30208372 PMC6583874

[143] Wang L Mu L Ren Z Tang H Wei Y Wang W Predictive role of executive function in the efficacy of intermittent theta burst transcranial magnetic stimulation modalities for treating methamphetamine use disorder—a randomized clinical trial. *Front Psychiatry.* (2021) 12:774192. 10.3389/fpsyt.2021.774192 34925101 PMC8674464

[144] Wen Y Li Y Jiang F Dong X. Tbs combined with virtual-reality reconsolidation intervention for methamphetamine use disorder: a pilot study. *Brain Stimul.* (2022) 15:996–8. 10.1016/j.brs.2022.07.001 35835436

[145] Liu Q Sun H Hu Y Wang Q Zhao Z Dong D Intermittent theta burst stimulation Vs. High-frequency repetitive transcranial magnetic stimulation in the treatment of methamphetamine patients. *Front Psychiatry.* (2022) 13:842947. 10.3389/fpsyt.2022.842947 35558419 PMC9087275

[146] Chen T Su H Li R Jiang H Li X Wu Q A transcranial magnetic stimulation protocol for decreasing the craving of methamphetamine-dependent patients. *STAR Protoc.* (2021) 2:100944. 10.1016/j.xpro.2021.100944 34825214 PMC8603307

[147] Yang J Li N Chen Y Sawan M editors. Towards Intelligent noninvasive closed-loop neuromodulation systems. *Proceedings of the 2022 IEEE 4th International Conference on Artificial Intelligence Circuits and Systems (AICAS).* Incheon: (2022).

[148] Li H Wang J Zhao S Tian F Yang J Sawan M editors. Real-time biosignal recording and machine-learning analysis system. *Proceedings of the 2022 IEEE 4th International Conference on Artificial Intelligence Circuits and Systems (AICAS).* Incheon: (2022).

[149] Chen C Tsai M Wu E Chung C Lee Y Chiu P Neuronal abnormalities induced by an intelligent virtual reality system for methamphetamine use disorder. *IEEE J Biomed Health Inform.* (2022) 26:3458–65. 10.1109/JBHI.2022.3154759 35226611

[150] Yang B Gu X Gao S Yan L Xu D Wang W. Different types of drug abusers prefrontal cortex activation patterns and based on machine-learning classification. *J Innov Opt Health Sci.* (2022) 15:2250012. 10.1142/S1793545822500122

